# Biofilm‐Mediated Persistence of Salmonella in Poultry Processing: A Comprehensive Review of Isolate Heterogeneity, Stress Adaptation, and Antimicrobial Resistance

**DOI:** 10.1111/1541-4337.70674

**Published:** 2026-10-12

**Authors:** Md Anamul Hasan Chowdhury, Chowdhury Sanat Anjum Reem, Md Furkanur Rahaman Mizan, Md. Ashrafudoulla, Sang‐Do Ha

**Affiliations:** ^1^ Department of Food Safety and Regulatory Science Chung‐Ang University Anseong Gyeonggi‐Do Republic of Korea; ^2^ Department of Food Science and Technology The Ohio State University Columbus Ohio USA; ^3^ GreenTech‐based Food Safety Research Group, BK21 Four Chung‐Ang University Anseong Gyeonggi‐Do Republic of Korea

**Keywords:** antimicrobial resistance, biofilm, food safety, poultry processing, Salmonella, stress adaptation

## Abstract

*Salmonella enterica* remains a major foodborne pathogen in poultry‐processing environments, where its persistence is largely attributed to biofilm formation on food‐contact and non‐food‐contact surfaces. Despite extensive research on *Salmonella* control, persistent contamination continues to challenge food safety due to the complex interplay among isolate‐specific traits, stress adaptation, and antimicrobial resistance (AMR). This review critically examines the phenotypic and genotypic determinants associated with Salmonella biofilm formation and evaluates their contributions to persistence, stress adaptation, and AMR. Emerging evidence also indicates that mixed‐species interactions, particularly with *Pseudomonas* spp., can influence biofilm architecture, stress tolerance, and the long‐term persistence of *Salmonella* in poultry‐processing environments. Particular attention is given to processing‐associated stresses, including temperature fluctuations, desiccation, nutrient limitation, and sublethal sanitizer exposure, which can induce adaptive responses that enhance biofilm development, promote persistence, and increase tolerance to antimicrobial interventions. The review further evaluates current detection approaches, including viability‐based quantification methods such as digital droplet PCR, and discusses their limitations and applications in identifying and quantifying biofilm‐associated and viable but nonculturable (VBNC) populations within processing environments. Existing control measures are assessed with emphasis on the challenges posed by mature biofilms and adaptive resistance mechanisms. Emerging antibiofilm technologies, including enzymatic treatments, bacteriophages, and UV‐LED systems, are also examined for their potential industrial implementation. By integrating current knowledge on biofilm biology, stress adaptation, and AMR, this review provides a framework for developing targeted and sustainable strategies to mitigate *Salmonella* persistence in poultry‐processing environments.

## 1 Introduction

*Salmonella enterica* is among the most common causes of foodborne illness worldwide, with chicken products consistently serving as a key reservoir and transmission vehicle (Chard et al. 2025). Despite significant advancements in hygiene procedures, regulatory monitoring, and processing technology, the frequency of *Salmonella*‐related outbreaks in poultry has not been successfully eliminated or significantly mitigated. This ongoing public health challenge highlights fundamental limitations in existing control measures, particularly within complex industrial systems where microbial contamination is difficult to eliminate (Chawla et al. 2023). A critical factor underlying this persistence is the organism's ability to form biofilms on diverse food‐contact surfaces. Biofilms are structured microbial communities embedded in a self‐produced extracellular polymeric substance (EPS) matrix, which confers both physical protection and physiological advantages to resident cells (Pang et al. 2023; Rossi et al. 2017). Surfaces in poultry‐processing facilities, such as stainless‐steel equipment, conveyor belts, and cutting instruments, provide ideal substrates for biofilm formation. Consequently, biofilm development is widely regarded as a major failure point in conventional cleaning and disinfection procedures.

The global burden of nontyphoidal Salmonella is estimated at approximately 93.8 million cases and 155,000 deaths annually (G. Kumar, Kumar, et al. 2025). Poultry and egg products account for a major portion of this burden, with multiyear epidemiological surveillance data demonstrating that between 20% and 25% of all foodborne *Salmonella* illnesses are directly attributed to the consumption of contaminated chicken and turkey products (Hammons 2024; USDA‐FSIS 2013). Within industrial poultry‐processing systems, the structural complexity of equipment, the continuous throughput of contaminated carcasses, and the repeated cycling of wet and dry conditions collectively create environments that are exceptionally permissive for biofilm establishment and long‐term persistence (S. H. Chen et al. 2020). Traditional sanitation methods, including the use of chemical disinfectants such as chlorine, peracetic acid (PAA), and quaternary ammonium compounds (QACs), are often ineffective against mature biofilms (Mendoza et al. 2022). The limited efficacy of these treatments is attributed to multiple factors, including poor antimicrobial penetration through the EPS matrix, reduced metabolic activity of biofilm‐associated cells, and the presence of highly resistant persister populations (Sharif and Yadav 2025). In addition, repeated exposure to sublethal concentrations of sanitizers in industrial environments can inadvertently induce adaptive responses that enhance both biofilm development and antimicrobial resistance (AMR) (Liao et al. 2020; Cadena et al. 2019). Conventional monitoring approaches, which mainly detect planktonic cells, frequently underestimate biofilm‐associated populations, leading to inadequate contamination risk assessments (Ban‐Cucerzan et al. 2025). Importantly, substantial variability among *Salmonella* isolates is an emerging and underexplored aspect of persistence. Not all isolates exhibit the same capacity to form biofilms, tolerate stress, or resist antimicrobials (Krüger et al. 2025; Bezek et al. 2023). Differences in genetic backgrounds, regulatory mechanisms, and prior environmental exposures contribute to isolate‐level diversity. Consequently, certain isolates, termed “high‐risk” or “persistent” strains, disproportionately account for long‐term contamination and recurrent outbreaks (R. A. Cheng, Eade, et al. 2019). However, current research and management strategies often overlook this heterogeneity, treating pathogen populations as functionally homogeneous. This gap is particularly consequential because persistence‐associated traits, including enhanced adhesion, robust EPS production, upregulated efflux pumps, and enhanced stress‐response genes, are not uniformly distributed across isolate populations but are concentrated within a minority of strains that disproportionately drive contamination recurrence.

Beyond the direct food safety consequences, persistent *Salmonella* contamination can impose substantial operational and economic burdens on poultry‐processing facilities. Recurrent contamination may necessitate intensified environmental monitoring, additional sanitation procedures, verification testing, product retention, and, in severe cases, temporary production interruptions, thereby increasing labor, water, energy, and chemical expenditures (Neelawala et al. 2024; Sidky et al. 2026). Persistent contamination may also contribute to product recalls, market withdrawals, regulatory scrutiny, and potential losses in consumer confidence and market access (Albaseer et al. 2026). Accordingly, persistent *Salmonella* contamination represents not only a food safety challenge but also an important consideration for operational sustainability and economic resilience within poultry‐processing systems. Despite growing recognition of these challenges, several critical knowledge gaps remain. First, there is a lack of comprehensive studies examining *Salmonella* isolates under conditions that closely mimic real‐world poultry‐processing environments, limiting the translational relevance of laboratory findings (Abrego et al. 2025). Second, biofilm formation, stress adaptation, and AMR are often investigated in isolation, resulting in a fragmented understanding of their collective impact on persistence (H. Y. Liu et al. 2024). Third, emerging antibiofilm strategies have not undergone adequate validation at the industrial scale, particularly concerning scalability, cost‐effectiveness, and regulatory compliance. Finally, the extent and impact of strain‐to‐strain variability on biofilm behavior and resistance phenotypes are not well characterized (Hindieh et al. 2025). Collectively, these gaps reflect a broader structural limitation: the field has advanced mechanistic understanding of individual persistence traits without yet producing integrated, isolate‐level frameworks capable of guiding precision‐based control in commercial environments.

Addressing these gaps is essential for developing effective and durable control strategies. This review integrates isolate‐level characterization with mechanistic insights into biofilm formation and stress adaptation and critically evaluates current and emerging translational approaches for controlling *Salmonella* in poultry processing. Unlike conventional reviews that examine biofilm formation, stress adaptation, or AMR independently, this work presents an integrated, isolate‐level framework linking these processes within the context of poultry‐processing environments. By emphasizing strain‐to‐strain heterogeneity and highlighting high‐risk persistent isolates, this review advances understanding beyond population‐based generalizations toward targeted risk assessment. Furthermore, the study bridges mechanistic insights and industrial applications, providing a systems‐level perspective to inform more effective and sustainable interventions. The overarching goal is to provide a translational roadmap connecting isolate heterogeneity, adaptive mechanisms, and resistance phenotypes with practical strategies for reducing persistent Salmonella contamination across the poultry‐processing continuum.

## 2 Literature Search Methodology

This review was developed through a structured literature search and narrative synthesis approach designed to comprehensively evaluate current knowledge on biofilm‐mediated *Salmonella* persistence in poultry‐processing environments. The search strategy was informed by established scoping review principles (Arksey and O'Malley 2005; Peters et al. 2020) and adapted to address the multidisciplinary nature of research spanning food microbiology, biofilm biology, stress adaptation, AMR, and poultry processing. These principles were used to support a structured and comprehensive approach to literature identification and thematic synthesis; however, the present study was designed as a structured narrative review and was not conducted as a formal scoping review requiring full PRISMA‐ScR reporting. Literature searches were conducted using PubMed, Web of Science, Scopus, and Google Scholar. The search was initially performed in December 2025 and subsequently updated in March 2026 to capture the most recent developments in the field. Search terms combined keywords related to *Salmonella*, biofilm formation, poultry processing, food‐contact surfaces, stress adaptation, AMR, disinfectant tolerance, and persistence. Boolean operators and database‐specific search functions were applied to maximize retrieval of relevant studies.

Studies were considered eligible if they investigated *Salmonella* serovars relevant to food production systems and addressed one or more aspects of biofilm formation, persistence, stress adaptation, AMR, environmental survival, detection, or control strategies. Particular emphasis was placed on research conducted in poultry production and processing environments or on food‐contact surfaces relevant to poultry operations. Peer‐reviewed articles published in English between January 2000 and March 2026 were considered, with special attention given to studies published during the last decade to reflect recent advances in the field. Publications lacking direct relevance to food processing environments, conference abstracts, book chapters, non‐peer‐reviewed literature, and studies focusing exclusively on planktonic behavior without consideration of biofilm‐associated characteristics were excluded. The retrieved records were screened for relevance based on their titles, abstracts, and, where necessary, full‐text assessment according to the predefined eligibility criteria. Because this review was designed as a structured narrative synthesis rather than a formal systematic or scoping review, a PRISMA‐ScR‐style quantitative accounting of the number of records identified, screened, and excluded was not prospectively maintained. Nevertheless, the literature selection process was consistently guided by the stated inclusion and exclusion criteria to ensure that the evidence incorporated into the review remained relevant to its predefined scope and objectives.

The retrieved literature was screened for relevance and evaluated according to its contribution to the major themes of this review. Information relating to isolate heterogeneity, biofilm characteristics, environmental adaptation, AMR, contamination ecology, and intervention strategies was systematically extracted and critically synthesized. The literature discussed in Sections 11–15 was identified through the same literature search and selection framework described above and was included when it directly addressed the thematic scope of the review. These sections therefore do not represent literature outside the scope of the reported search; rather, they synthesize evidence identified through the same overall literature search and organized according to the major themes of the review. Owing to substantial variation in study objectives, experimental methodologies, analytical approaches, and reported outcomes, a narrative synthesis framework was adopted rather than a quantitative meta‐analysis. This approach enabled the integration of evidence across diverse study designs while facilitating a comprehensive evaluation of the mechanisms underlying biofilm‐mediated persistence of *Salmonella* in poultry‐processing environments.

## 3 *Salmonella* Serovars of Concern in Poultry

The genus *S. enterica* includes more than 2600 serovars, yet only a small number are responsible for most human infections linked to poultry (Grimont and Weill 2007; Marshall et al. 2024). Understanding which serovars dominate in different regions, how strongly they form biofilms, and where new threats are emerging is essential for designing effective control measures.

### 3.1 Dominant Serovars in Poultry

The epidemiology of Salmonella in poultry is characterized by the recurrent dominance of a limited number of serovars, although their prevalence and public health significance vary considerably across geographic regions. Among the numerous serovars identified in poultry production systems, Salmonella Enteritidis, Typhimurium, Infantis, and Kentucky are consistently reported as the most prevalent and epidemiologically relevant worldwide (Willson and Chousalkar 2023). Their continued circulation within poultry production systems reflects a combination of ecological fitness, adaptation to farm and processing environments, and, in some cases, enhanced virulence and AMR traits. Regional surveillance programs reveal notable differences in serovar distribution. In the United States, S. Kentucky and S. Infantis are frequently recovered from broiler carcasses and poultry products. Despite its widespread occurrence, S. Kentucky is only infrequently associated with human salmonellosis, highlighting the important distinction between prevalence within poultry systems and actual public health risk (Georganas et al. 2024; M. Li, Barnett‐Neefs, et al. 2026). In contrast, S. Infantis has emerged as a major food safety concern because of its increasing association with AMR, enhanced virulence capabilities often mediated by plasmid‐borne pathogenicity factors that facilitate cellular adhesion and systemic colonization, and its capacity to persist throughout poultry production and processing environments (Wenger et al. 2017; Drauch et al. 2021).

Evidence from longitudinal investigations further demonstrates that certain serovars possess a selective advantage during processing. A large‐scale study tracking Salmonella dissemination across multiple broiler production complexes in the United States found that S. Enteritidis, S. Typhimurium, and S. Infantis collectively represented a disproportionately high proportion of isolates recovered from carcasses and retail chicken parts compared with their representation in breeder flocks (Gu et al. 2019). This observation suggests that these serovars are selectively maintained or exhibit prolonged environmental persistence throughout processing operations, rather than undergoing active metabolic enrichment. This localized success is heavily driven by their elevated tolerance to environmental stressors, superior surface attachment kinetics, and heightened resistance to routine chemical cleaning and sanitation regimens (Oliveira et al. 2026). Such characteristics may facilitate survival along the farm‐to‐fork continuum and contribute to their recurring involvement in foodborne outbreaks (T. Kim et al. 2025).

Interpretation of serovar prevalence data should also consider potential methodological biases associated with enrichment and isolation procedures used in foodborne pathogen testing. Selective enrichment broths, plating media, and recovery protocols may differentially support the growth and detection of specific *Salmonella* serovars, thereby influencing recovery frequencies and surveillance outcomes (Gorski et al. 2024). Consequently, observed serovar distributions may reflect not only ecological fitness, persistence, and transmission dynamics but also, to some extent, differences in the efficiency with which individual serovars are recovered by commonly used detection methods. This consideration is particularly important when comparing prevalence estimates across studies, regions, and surveillance programs that may employ different analytical methodologies (Siceloff et al. 2025). The dominant serovars identified in poultry also vary substantially across different regions of the world. In Egypt, S. Enteritidis remains the predominant serovar recovered from broiler production systems, whereas the detection of S. enterica subsp. Salamae has highlighted the presence of less commonly reported poultry‐associated serovars (Shaheed et al. 2026). Similarly, investigations in Nigeria have identified S. Typhimurium as the leading serovar, followed by S. Enteritidis and S. Anatum, indicating substantial regional heterogeneity in serovar composition and transmission dynamics (Fadipe and Hölzle 2025). These differences likely reflect variations in poultry management practices, biosecurity measures, environmental conditions, and local antimicrobial usage patterns.

Within Europe, a notable epidemiological shift has occurred in recent years, with S. Infantis surpassing several traditionally dominant serovars to become the most frequently detected serovar in broiler meat. This emergence has been largely attributed to the dissemination of the pESI megaplasmid, a mobile genetic element that confers multiple adaptive advantages, including enhanced biofilm formation, increased AMR, and improved adhesion to both host tissues and food‐contact surfaces (EFSA and ECDC 2025). The success of *S*. Infantis illustrates how the acquisition of mobile genetic elements can rapidly alter the epidemiological landscape of *Salmonella* within poultry production systems. The emergence of *S*. Infantis has also been accompanied by increasing concern regarding its exceptional persistence and AMR. In addition to its widespread dissemination in broiler production systems, *S*. Infantis exhibits strong biofilm‐forming capacity and enhanced environmental resilience, traits that have been linked in part to the pESI megaplasmid and its associated persistence, adhesion, and resistance determinants. Recent genomic surveillance has further identified mcr‐1‐positive *S*. Infantis isolates from broiler production systems, highlighting the emergence of plasmid‐mediated colistin resistance in this serovar and reinforcing concerns regarding the convergence of biofilm‐associated persistence and clinically important AMR (Habib et al. 2025).

Another emerging serovar of concern is *S*. Minnesota, which has demonstrated remarkable environmental persistence in poultry slaughterhouses. Recent studies have shown that *S*. Minnesota can maintain substantial biofilm formation under refrigeration conditions, including temperatures commonly encountered during chilling operations, indicating a cold‐adaptive phenotype capable of circumventing the protective effects typically associated with refrigeration. The presence of biofilm‐associated genes together with frequent AMR determinants further supports the role of *S*. Minnesota as a potentially persistent reservoir within poultry‐processing environments (Justino et al. 2026). These observations strengthen the concept that the epidemiological success of certain poultry‐associated serovars is driven by the interconnected effects of stress adaptation, biofilm formation, and AMR, rather than by prevalence alone.

Collectively, these findings indicate that the dominance of specific *Salmonella* serovars is not solely determined by their prevalence in poultry populations but also by their ability to persist under production‐associated stresses, colonize processing environments, and survive intervention measures. Understanding the biological and ecological factors that underpin serovar‐specific persistence is therefore essential for developing targeted control strategies aimed at reducing *Salmonella* contamination and mitigating foodborne disease risk. While the prevalence of individual serovars is important for understanding contamination patterns, persistence within poultry environments is also strongly influenced by their capacity to form biofilms. Consequently, evaluating serovar‐specific differences in biofilm development is critical for understanding long‐term contamination dynamics.

### 3.2 Serovar‐Specific Differences in Biofilm Formation

The ability to form biofilms is a defining trait that contributes to the persistence of *Salmonella* within poultry production and processing environments (Castelijn et al. 2013). However, biofilm formation is not a uniform characteristic across the genus. Considerable heterogeneity exists not only among different serovars but also among isolates belonging to the same serovar, reflecting the complex interplay of genetic background, regulatory networks, and environmental adaptation (Castelijn et al. 2013). This variability has important implications for contamination persistence, transmission dynamics, and the efficacy of sanitation interventions. Comparative investigations involving major poultry‐associated serovars, including *S*. Typhimurium, Infantis, Derby, and Brandenburg, have consistently demonstrated substantial differences in biofilm‐forming capacity (Edison and Kariyawasam 2026). Using standardized crystal violet microtiter assays conducted on polystyrene surfaces, researchers observed that biofilm biomass production was highly variable; under identical incubation conditions (e.g., 20°C–25°C representing processing‐plant ambient temperatures in nutrient‐limited media), some isolates produced exceptionally dense, mature structures (quantified by an optical density [OD] at 570 or 595 nm; OD≥1.0), while others generated negligible biomass (OD<0.1) (Bashir et al. 2019). Notably, the greatest phenotypic diversity and heterogeneity have been reported among *S*. Typhimurium and *S*. Infantis isolates, with several studies demonstrating that even closely related strains within these serovars exhibit drastically different attachment kinetics and final biomass yields (S.‐H. Kim et al. 2022). This confirms that biofilm development is highly strain‐dependent even within epidemiologically important serovars. Such findings highlight the limitations of predicting persistence potential solely on the basis of serovar identity and underscore the importance of considering intra‐serovar variation when evaluating contamination risks (Yogendraiah et al. 2026).

The influence of serovar‐specific traits becomes particularly evident on food‐processing surfaces. Stainless steel, one of the most widely used materials in poultry‐processing facilities, serves as a critical interface for bacterial attachment and biofilm establishment. Comparative analyses have revealed that the biofilm behavior of *S*. Typhimurium is often more closely related to that of *S*. Infantis than to *S*. Derby or *S*. Brandenburg, suggesting the existence of shared genetic determinants or regulatory pathways that govern surface colonization and biofilm maturation (Edison and Kariyawasam 2026). These similarities may help explain the frequent association of *S*. Typhimurium and *S*. Infantis with persistent contamination in poultry‐processing environments, although persistence cannot be attributed to serovar identity alone. Biofilm formation provides a significant ecological advantage by enhancing bacterial survival under adverse environmental conditions. Cells embedded within the extracellular polymeric matrix generally exhibit greater resistance to desiccation, nutrient limitation, and antimicrobial interventions than their planktonic counterparts (Yaki 2026). Consequently, biofilm‐associated *Salmonella* populations can persist on processing surfaces for extended periods, serving as reservoirs for recurrent contamination. Nevertheless, the protective effect of biofilms is not absolute. Studies evaluating PAA, one of the most commonly used sanitizers in poultry processing, have reported only modest increases in resistance among biofilm‐associated cells. Moreover, the presence of organic matter can substantially reduce sanitizer efficacy, thereby limiting sanitizer penetration and activity, while the biofilm matrix may continue to provide additional protection to embedded cells against residual sanitizer exposure (Chapagain et al. 2026). These observations emphasize the importance of evaluating sanitation strategies under realistic processing conditions, where residual organic material can profoundly influence antimicrobial performance.

In commercial poultry‐processing environments, *Salmonella* frequently exists within mixed‐species biofilms rather than as a single‐species population. Emerging evidence indicates that interactions with resident microorganisms, particularly *Pseudomonas* spp., can substantially influence biofilm architecture, extracellular matrix production, nutrient utilization, and tolerance to environmental stresses and antimicrobial interventions (Xu et al. 2025; D. Wang, Wang, Liu, et al. 2025). In addition, mixed‐species biofilms may modify the susceptibility of Salmonella to sanitizers by altering extracellular matrix composition, metabolic interactions, and spatial organization within the biofilm. These complex microbial communities also provide opportunities for horizontal gene transfer (HGT) and the dissemination of AMR determinants, further contributing to the persistence and resilience of Salmonella in poultry‐processing environments (Magdalena et al. 2026). Thus, serovar‐ and strain‐specific characteristics should be interpreted within the broader ecological context of mixed‐species biofilms, in which interactions among microorganisms can modify the phenotype of individual populations. These interspecies interactions may promote the establishment of more structured and resilient biofilm communities, thereby facilitating long‐term environmental persistence, survival following sanitation procedures, and recurrent contamination events. Consequently, the persistence of Salmonella in poultry‐processing environments should be regarded as an emergent property of isolate‐specific phenotypes, environmental conditions, and community‐level ecological interactions, rather than as a characteristic of individual isolates alone (Adeyemi and Nahashon 2026). Quantitative assessments further demonstrate that biofilm accumulation varies according to both serovar and surface characteristics. On stainless steel, *S*. Typhimurium commonly reaches populations exceeding 10^5^–10^6^ CFU/cm^2^ within the first 24–48 h, with cell densities continuing to increase during prolonged incubation (Białucha et al. 2020). Similar trends have been reported on plastic‐based materials, whereas rougher substrates such as concrete and ceramic surfaces often support even greater bacterial accumulation. In several studies, biofilm populations on ceramic tiles exceeded 10^7^ CFU/cm^2^ after incubation periods ranging from 5 to 14 days, depending on the experimental conditions, highlighting the capacity of porous or irregular surfaces to facilitate bacterial retention and biofilm maturation (Featherstone et al. 2023; Zhang et al. 2025). Comparable patterns have also been observed for *S*. Enteritidis, which frequently attains bacterial densities similar to or greater than those of *S*. Typhimurium on food‐contact and environmental surfaces. Although surface physicochemical properties are frequently considered key determinants of bacterial attachment, their role remains complex and sometimes contradictory. Several investigations have reported enhanced attachment of *Salmonella* to hydrophobic materials, suggesting that surface hydrophobicity may promote initial adhesion (Rocha et al. 2026). For example, greater recovery of *S*. Enteritidis has been observed on hydrophobic substrates than on hydrophilic stainless‐steel surfaces. Similarly, differences in bacterial recovery have been documented among various plastic materials, including polyvinyl chloride and polyethylene. However, other studies have found minimal or no significant differences in attachment and biofilm formation across commonly used food‐contact materials (Counihan et al. 2025). Under controlled experimental conditions, *S*. Enteritidis and *S*. Typhimurium have demonstrated comparable colonization of stainless steel, plastic, silicone rubber, and polypropylene surfaces, indicating that bacterial physiological traits may exert a stronger influence on biofilm development than surface composition alone (Gómez et al. 2016).

The importance of serovar‐specific differences is further illustrated by *S*. Minnesota, a serovar that has been recognized for decades but has received increasing attention in recent poultry‐associated surveillance and persistence studies. Rather than representing a newly emerged serovar, *S*. Minnesota is increasingly recognized as a relevant poultry‐associated lineage because of its reported occurrence in production systems and its capacity to persist under processing‐associated conditions (Brasão et al. 2021). Several studies have identified biofilm‐associated determinants, including *csgD* and *adrA*, in *S*. Minnesota, while AMR determinants, including extended‐spectrum β‐lactamase (ESBL)‐encoding genes, have also been reported in some isolates (Justino et al. 2026). A particularly relevant characteristic of *S*. Minnesota is its reported ability to maintain biofilm formation under refrigeration conditions. Studies have indicated that this serovar can retain measurable biofilm‐forming capacity at temperatures relevant to poultry chilling operations, including 4°C–8°C, although the magnitude of biofilm formation is dependent on strain and experimental conditions (Justino et al. 2026; Counihan et al. 2025). Such cold‐associated persistence may provide an ecological advantage during chilling and subsequent processing stages, where low temperatures can constrain the growth of many microorganisms but may not completely prevent biofilm development or survival.

Molecular epidemiological investigations have further reported genetically related *S*. Minnesota strains across carcasses, cecal contents, and chiller water over extended sampling periods, suggesting the potential establishment and maintenance of resident populations within slaughterhouse environments rather than repeated independent introduction alone (Justino et al. 2026). When considered together, these observations indicate that the importance of *S*. Minnesota lies not in its recent origin, but in its increasing recognition as a poultry‐associated serovar capable of combining biofilm formation, environmental persistence, cold‐associated survival, and AMR in some isolates. This example further demonstrates why serovar identity should be considered alongside strain‐level characteristics, environmental conditions, surface properties, and microbial community interactions when assessing the persistence of *Salmonella* in poultry‐processing environments.

Collectively, current evidence suggests that biofilm formation in Salmonella is governed by a combination of serovar‐specific characteristics, strain‐level variability, surface properties, and environmental factors. While surface properties can influence attachment and subsequent biofilm development, the intrinsic biological traits of individual isolates frequently determine the extent of persistence and resistance within poultry‐processing environments. Importantly, the relative contribution of these factors is context‐dependent, and no single characteristic can reliably predict persistence across all processing conditions. Understanding these serovar‐dependent and strain‐dependent differences is therefore essential for designing targeted intervention strategies capable of controlling persistent Salmonella contamination throughout the poultry production continuum. Although S. Enteritidis, S. Typhimurium, and S. Infantis remain among the major poultry‐associated serovars, the increasing recognition of S. Minnesota and other serovars with potentially enhanced persistence traits highlights the need for surveillance and intervention strategies that extend beyond the historically dominant serovars. Figure 1 summarizes the major poultry‐associated *Salmonella* serovars and highlights the key biological and ecological traits associated with persistence in poultry production and processing environments, including biofilm formation, stress adaptation, AMR, cold‐associated survival, and environmental persistence.

**FIGURE 1:**
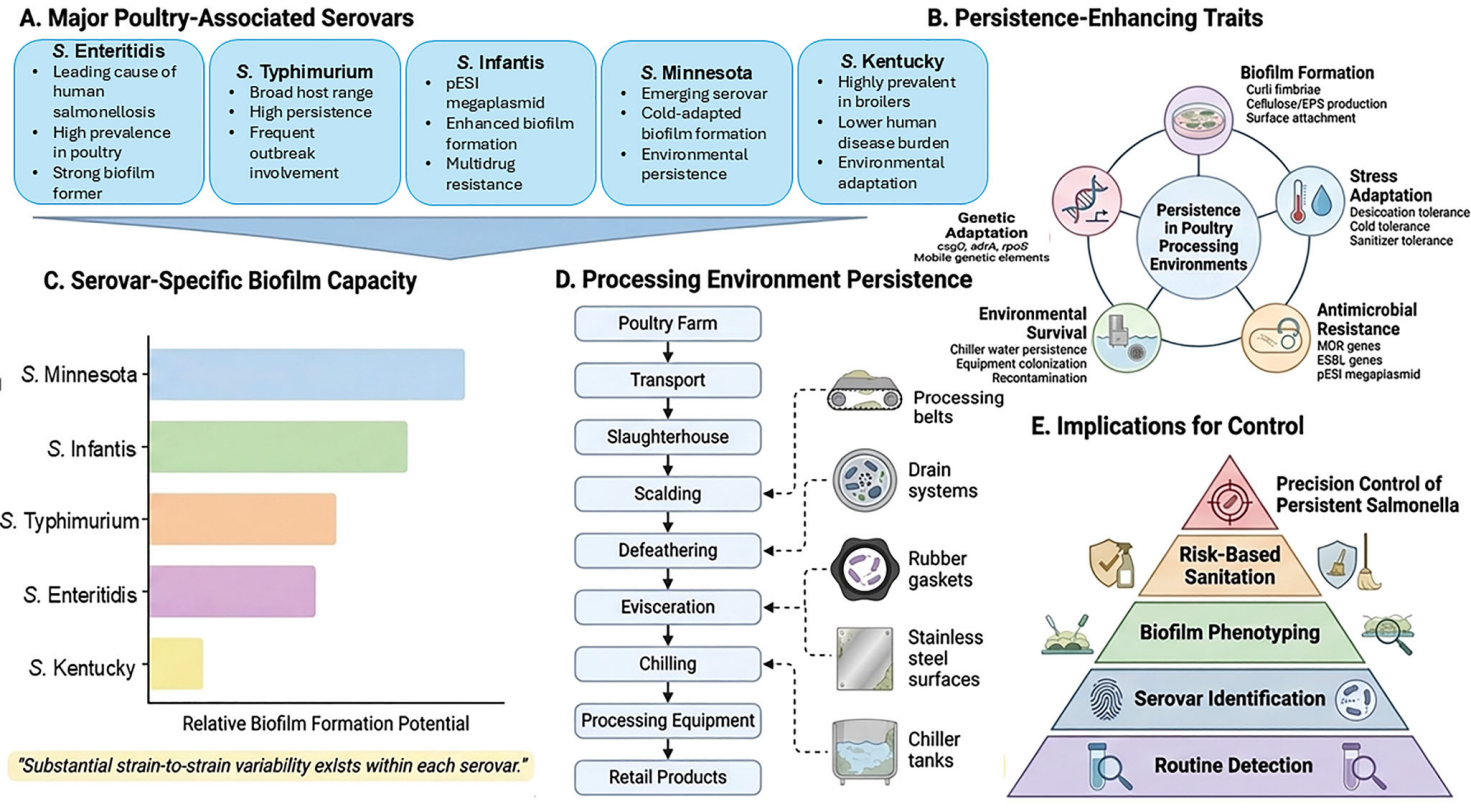
Serovar‐specific determinants of persistence and control challenges in poultry production systems. Major poultry‐associated serovars (*Salmonella* Enteritidis, Typhimurium, Infantis, Kentucky, and the emerging serovar Minnesota) differ in their biofilm‐forming capacity, stress adaptation mechanisms, antimicrobial resistance traits, and environmental persistence. These characteristics influence survival throughout poultry‐processing environments and contribute to recurrent contamination events. The figure illustrates the relationship between serovar‐specific persistence determinants, biofilm development on food‐contact surfaces, contamination hotspots, and the need for risk‐based control strategies targeting persistent *Salmonella* populations.

## 4 Ecology and Transmission Dynamics of Salmonella Isolates

The ecology and transmission of *Salmonella* within poultry systems are shaped by a complex continuum spanning farm production, slaughter, processing, and retail distribution. At the farm level, *Salmonella* colonizes the gastrointestinal tract of poultry and persists in litter, feed, water systems, and housing environments (Marcu et al. 2026a). These reservoirs represent important sources of contamination and contribute to the genetic and phenotypic diversity of isolates entering the processing chain. The persistence of Salmonella within farm‐associated reservoirs facilitates continual introduction of diverse populations into downstream processing environments, thereby influencing subsequent contamination dynamics. During transportation and preslaughter handling, stressors such as crowding and temperature fluctuations can further influence microbial shedding and dissemination (Gržinić et al. 2023). The transition from farm to slaughterhouse serves as a critical amplification point for contamination. Steps such as defeathering and evisceration facilitate the release and spread of intestinal contents, resulting in widespread surface contamination (Laconi et al. 2025). These processing operations are consistently recognized as major contamination points where cross‐contamination can occur between carcasses, equipment surfaces, and the surrounding environment. As carcasses progress through processing lines, including washing, chilling, deboning, and further processing operations, *Salmonella* can attach to and persist on a range of food‐contact surfaces. Materials such as stainless steel, polyethylene, and rubber provide optimal substrates for microbial adhesion and subsequent biofilm formation (Ivers et al. 2024). The establishment of surface‐associated populations enables long‐term environmental persistence and creates contamination reservoirs capable of seeding recurrent contamination events throughout processing facilities.

A primary driver of isolate diversity in these environments is the array of selection pressures encountered along the processing continuum. Environmental factors, such as temperature variation, moisture availability, nutrient limitation, and exposure to sanitizers, serve as selective forces shaping the survival and dominance of specific *Salmonella* populations. Repeated exposure to sublethal stress can favor isolates with enhanced stress tolerance, biofilm‐forming capacity, and AMR (Obe et al. 2024). Consequently, the ecological success of Salmonella is not determined solely by serovar prevalence but also by the adaptive characteristics that enable survival under production‐associated stresses. Over time, these selective pressures can favor resilient subpopulations with enhanced stress tolerance and persistence, facilitating their long‐term establishment as resident strains within food‐processing environments (Mkangara 2023).

Importantly, not all *Salmonella* isolates contribute equally to contamination and persistence. Emerging evidence highlights the existence of isolates with pronounced biofilm‐forming and stress‐tolerance phenotypes that display superior adhesion, robust biofilm architecture, and increased resistance to environmental and chemical stresses (Krüger et al. 2025). Biofilm populations on food‐contact surfaces can exceed 10^5^–10^7^ CFU/cm^2^ under favorable conditions, providing substantial protection against environmental challenges and sanitation procedures (Gharat et al. 2025). These isolates are frequently implicated in recurrent contamination events, as they establish stable biofilms in hard‐to‐clean niches such as conveyor belts, equipment joints, rubber gaskets, drains, and chiller systems and continuously release cells into the processing environment. The repeated release of cells from these persistent reservoirs can promote recontamination of carcasses and processing equipment, thereby sustaining transmission within the processing environment and potentially increasing the likelihood of downstream contamination. Identifying and characterizing these high‐risk isolates is therefore critical for understanding transmission pathways and prioritizing contamination reservoirs for targeted surveillance (Castello et al. 2025). Table 1 summarizes the primary contamination sites throughout the processing continuum, along with associated surface types, dominant isolate traits, and relative risk levels, providing a concise framework for understanding how environmental context shapes isolate behavior and persistence.

**TABLE 1:** Key stages, sites, surface types, isolate traits, biofilm and antimicrobial resistance (AMR) profiles, and relative risk assessment for Salmonella contamination across the poultry‐processing continuum.

Stage	Site	Surface	Key traits	Biofilm	AMR	Risk	References
Farm	Litter, feed, water	Organic	Diverse, adaptive	Moderate	Low–Moderate	Medium	Khalefa et al. 2025
Preslaughter	Crates, holding	Plastic, metal	Stress‐tolerant	Moderate	Moderate	High	Koutsoumanis et al. 2022
Slaughter	Defeathering, scalding	Metal, rubber	Heat‐tolerant, adherent	Moderate–High	Moderate	High	Adel et al. 2025
Evisceration	Gut‐contact surfaces	Steel, plastic	Strong adhesion; mixed‐species contamination	High	Moderate–High	Very High	Barbieri 2020
Processing	Conveyors, cutters	Steel, polyethylene	Strong biofilm, EPS‐rich; mixed‐species community interactions	Very High	High	Critical	Ban‐Cucerzan et al. 2025
Chilling	Water systems	Liquid‐contact	Cold‐tolerant; mixed‐species community interactions	High	Moderate–High	High	X. Wang, Zhao, et al. 2023
Postprocessing	Cutting areas	Mixed	Cross‐contaminating; mixed‐species community interactions	High	High	Very High	El Kadri et al. 2021
Retail	Packaging	Plastic	Stress‐resistant	Moderate	Moderate	Medium	Rather et al. 2021

*Note*: Biofilm refers to the relative level of biofilm‐forming capacity or biofilm persistence reported for *Salmonella* at the indicated stage or site based on the available literature. AMR, antimicrobial resistance, refers to the relative antimicrobial resistance potential or resistance profile reported for *Salmonella* populations at each stage. Risk represents a qualitative relative assessment of the potential for *Salmonella* contamination, persistence, and transmission at the indicated stage, considering biofilm‐associated persistence, antimicrobial resistance, environmental conditions, and opportunities for cross‐contamination. The descriptors “Low,” “Low–Moderate,” “Moderate,” “Moderate–High,” “High,” “Very High,” and “Critical” represent qualitative relative categories rather than quantitative or probabilistic risk estimates and were assigned based on the overall evidence synthesized from the cited literature.

However, current monitoring systems often fail to adequately capture the persistence and adaptive behavior of these resident strains, particularly when they occur at low abundance or exhibit stress‐induced phenotypic changes (Cheng et al., 2019; G. Cheng, Ning, et al. 2019). Moreover, conventional surveillance approaches may not readily distinguish transient contamination from populations capable of establishing long‐term environmental reservoirs. As a result, isolates with markedly different persistence potential are frequently treated equivalently despite their substantially different implications for contamination risk and food safety outcomes (Pettengill et al. 2022). Integrating isolate‐level characterization with ecological and transmission data will be essential for identifying persistent reservoirs, resolving contamination pathways, and improving risk‐based surveillance throughout poultry‐processing systems (Chandross‐Cohen et al. 2025).

## 5 Characterization of Biofilm‐Forming *Salmonella* Isolates

Understanding the persistence of *Salmonella* in poultry‐processing environments requires a shift from population‐level generalizations to isolate‐level characterization. Growing evidence indicates that *Salmonella* isolates exhibit substantial heterogeneity in their ability to form biofilms, tolerate environmental stresses, and resist antimicrobial interventions (Betiku et al. 2025). This variability is a critical determinant of contamination dynamics, yet it remains insufficiently addressed in current research and control strategies. Comprehensive characterization encompassing both phenotypic traits and genotypic determinants is therefore essential for identifying high‐risk isolates and informing targeted mitigation strategies (He et al. 2026).

### 5.1 Phenotypic Characterization

Phenotypic characterization provides direct insight into the functional behavior of *Salmonella* isolates under conditions relevant to food processing environments. Among these traits, biofilm biomass variability is one of the most widely studied indicators, typically assessed using crystal violet staining or similar assays (Desmousseaux et al. 2025). Notably, significant differences are often observed among isolates, with some exhibiting weak or transient biofilm formation and others developing dense, highly structured communities. These differences have direct implications for persistence, as high‐biomass biofilms are more resistant to cleaning and disinfection procedures (Sakalauskienė and Radzevičienė 2025). Auto‐aggregation capacity is another important trait associated with biofilm formation. Isolates with strong auto‐aggregation tendencies can rapidly form microcolonies, facilitating early‐stage biofilm development (Tavaddod et al. 2024). This feature is closely linked to cell–cell adhesion mechanisms and can influence the speed and stability of biofilm establishment on food‐contact surfaces.

Cell surface hydrophobicity also plays a critical role in initial adhesion to abiotic surfaces such as stainless steel and plastic. Isolates with higher hydrophobicity generally demonstrate enhanced attachment efficiency, particularly under low‐nutrient or dynamic flow conditions commonly encountered in processing environments (Yücetepe et al. 2026). However, the relationship between hydrophobicity and biofilm formation is not always linear, indicating the involvement of additional regulatory factors. Motility, particularly flagella‐mediated movement, contributes to both surface colonization and biofilm architecture (Romero et al. 2025). Motile isolates can more effectively explore and colonize surfaces, while also influencing the spatial organization of biofilms. In some cases, however, reduced motility has been associated with increased biofilm stability, reflecting a trade‐off between dispersal and persistence (J. Wang, Hashem, et al. 2025).

Collectively, these phenotypic traits highlight the functional diversity among *Salmonella* isolates and underscore the need for multiparameter characterization approaches rather than reliance on single indicators.

### 5.2 Genotypic Characterization

At the molecular level, biofilm formation and persistence are orchestrated by a complex network of genes and regulatory pathways. Adhesion‐related genes, including those encoding fimbriae and flagellar components, play a central role in mediating initial surface attachment (Bundurus et al. 2024). Variations in the presence, expression, or regulation of these genes can substantially influence the adhesive capacity of different isolates. EPS‐related genes direct the synthesis of key biofilm matrix components, such as polysaccharides, proteins, and extracellular DNA (eDNA) (Zhao et al. 2023). These matrix constituents not only confer structural integrity but also contribute to antimicrobial tolerance by restricting disinfectant penetration. Differences in EPS composition and production among isolates can thus produce distinct biofilm architectures and resistance profiles (Blaznik et al. 2025).

Stress‐response genes are essential for survival under the harsh conditions encountered during poultry processing, such as temperature fluctuations, desiccation, and exposure to sanitizers. Activation of these genes can enhance both biofilm formation and resistance, particularly by inducing protective pathways and metabolic adjustments (Marmion et al. 2022). In addition, AMR determinants, including genes encoding efflux pumps and resistance enzymes, are frequently enhanced in biofilm‐forming isolates. The close association between biofilm formation and AMR suggests these traits may be co‐regulated or co‐selected under processing conditions (H. Y. Liu et al. 2024). A key challenge in interpreting isolate behavior lies in the disconnect between genotype and phenotype. The mere presence of biofilm‐associated or resistance genes does not necessarily equate to functional expression, as gene regulation is highly context‐dependent and shaped by environmental conditions (Nour El‐Din et al. 2021). This regulatory complexity frequently leads to inconsistencies between predicted and observed behaviors, complicating risk assessment and intervention strategies. Moreover, the substantial variability among *Salmonella* isolates underscores that persistence and resistance are not uniform traits (Lamichhane et al. 2024). Rather, these characteristics are unevenly distributed across populations, with a subset of high‐risk isolates displaying disproportionately elevated biofilm‐forming capacity and stress tolerance. Such high‐risk isolates are likely to play a dominant role in long‐term contamination and recurrent outbreaks (Musa et al. 2024). The integrated relationships between phenotypic traits, genotypic determinants, and environmental stressors that drive biofilm formation and persistence in *Salmonella* are illustrated in Figure 2. Collectively, these findings highlight the genetic basis of isolate‐level variation in biofilm formation, stress tolerance, and AMR, while the specific mechanisms through which biofilm development alters antimicrobial susceptibility are discussed separately in Section 7.

**FIGURE 2:**
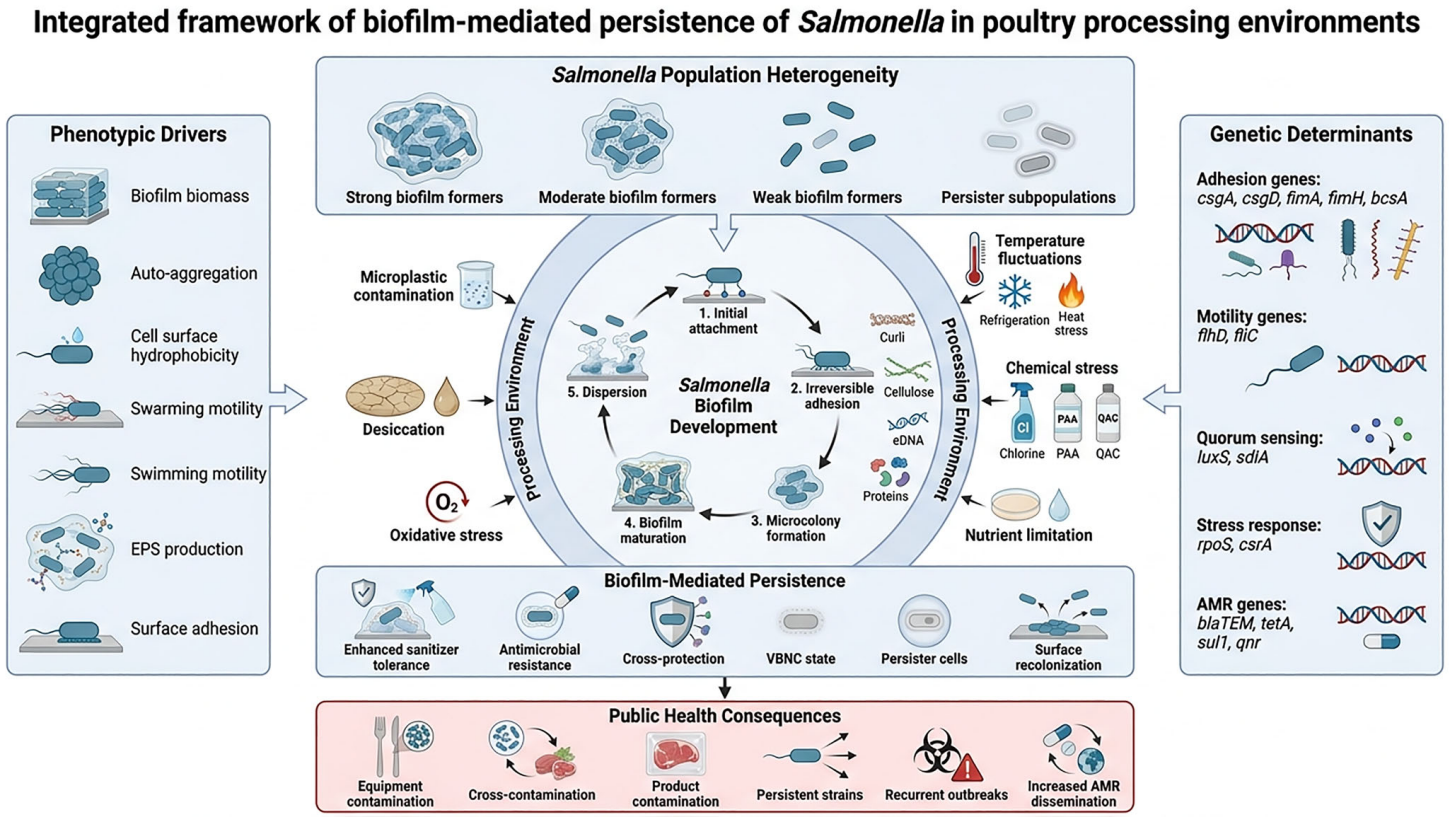
Integrated framework of biofilm formation and persistence in Salmonella. Phenotypic characteristics, including biofilm biomass, auto‐aggregation, cell surface hydrophobicity, and motility, interact with genetic determinants such as adhesion‐associated genes, extracellular polymeric substance (EPS) biosynthesis pathways, stress‐response regulators, and antimicrobial resistance (AMR) genes. These factors converge to drive biofilm development, resulting in enhanced persistence and resistance under processing conditions. Environmental stresses (temperature fluctuations, sanitizer exposure, nutrient limitation) further modulate these interactions, promoting the emergence of high‐risk isolates associated with persistent contamination and recurrent outbreaks. Overall, the figure provides a systems‐level overview of how phenotypic traits, genetic determinants, and environmental stressors collectively drive biofilm formation and persistence in Salmonella within poultry‐processing environments.

### 5.3 Correlation Between Phenotypic and Genotypic Traits

Phenotypic and genotypic characterization provide complementary perspectives on the persistence potential of *Salmonella* isolates; however, neither approach alone is sufficient to accurately predict behavior in poultry‐processing environments. Although numerous studies have identified associations between biofilm‐related genes and persistence‐associated phenotypes, the relationship between genotype and phenotype is often inconsistent and highly context‐dependent (Justino et al. 2026). In particular, the detection of adhesion‐, EPS‐, stress‐response‐, and AMR‐associated genes does not necessarily correspond to the magnitude of biofilm formation, stress tolerance, or persistence observed under processing‐relevant conditions.

This variability reflects the complex regulatory networks governing biofilm development and environmental adaptation (Yaki 2026). Isolates harboring similar complements of persistence‐associated genes frequently exhibit markedly different phenotypic profiles, indicating that gene expression, regulatory interactions, and environmental stimuli collectively determine functional outcomes. Factors such as nutrient availability, temperature fluctuations, surface characteristics, and sanitizer exposure can substantially influence the expression of biofilm‐associated pathways, thereby shaping the extent of surface colonization, biofilm maturation, and survival under processing conditions (Zeng et al. 2025). Recent advances in transcriptomics, proteomics, comparative genomics, and integrated multiomics analyses have further demonstrated that persistence is a dynamic phenotype resulting from interactions among genetic determinants, regulatory mechanisms, and environmental pressures. These studies increasingly support the view that biofilm formation, stress adaptation, and AMR should not be considered independent traits but rather interconnected components of a broader persistence network that collectively influences isolate survival and contamination potential (Vohra et al. 2024).

Consequently, comprehensive characterization strategies that integrate phenotypic measurements with molecular and functional analyses provide the most robust framework for identifying high‐risk isolates and predicting their behavior within poultry‐processing environments. Such systems‐level approaches are expected to improve persistence risk assessment and support the development of more targeted and effective control strategies for *Salmonella*.

### 5.4 Identification of High‐Risk Isolates

The substantial heterogeneity among *Salmonella* isolates highlights the need to move beyond simple pathogen detection toward the identification of populations with the greatest persistence potential. Increasing evidence indicates that recurrent contamination events are often driven by a limited subset of highly adapted isolates capable of sustained survival under processing‐associated stresses (Hussain et al. 2025). These high‐risk isolates typically exhibit enhanced biofilm formation, increased stress tolerance, and elevated AMR, enabling their persistence within processing environments and contributing disproportionately to contamination recurrence. Identification of high‐risk isolates requires an integrated assessment of persistence‐associated phenotypes and genotypes rather than reliance on individual markers (Muteeb et al. 2025). Isolates displaying strong biofilm‐forming capacity together with multiple stress‐adaptation and AMR determinants are generally considered more likely to establish persistent environmental reservoirs than isolates lacking these characteristics. Such observations support the development of risk‐based characterization frameworks that evaluate persistence as a multifactorial trait arising from the interaction of biofilm formation, environmental adaptation, and resistance mechanisms (Roy et al. 2022). Consistent with the European Food Safety Authority (EFSA) assessment, current evidence does not support the existence of universal genetic markers that can reliably distinguish persistent from nonpersistent *Salmonella* strains across all production environments; therefore, persistence should be evaluated using integrated phenotypic, genomic, and ecological information (Koutsoumanis et al. 2024).

Incorporating these approaches into food safety monitoring frameworks could improve the early identification of high‐risk populations and facilitate more targeted interventions against persistent *Salmonella* contamination in poultry‐processing environments.

## 6 Molecular and Structural Basis of Biofilm Formation

Building on the genotypic determinants discussed in Section 5.2, a mechanistic understanding of biofilm formation in *Salmonella* is essential to explain how genetic potential is translated into phenotypic traits that support persistence in poultry‐processing environments. Biofilm development is governed by coordinated molecular processes that regulate surface attachment, matrix assembly, and community organization, ultimately determining structural stability and resistance phenotypes (X. Wang, Liu, et al. 2023). Initial adhesion is mediated by surface appendages, such as flagella and fimbriae, which facilitate attachment to abiotic substrates under dynamic conditions. These appendages enable both reversible and irreversible interactions with surfaces, influenced by physicochemical properties such as surface energy and roughness (Pal and M. Lavanya 2022). After attachment, cells transition to a sessile state and initiate matrix production, marking the onset of biofilm maturation. The EPS matrix constitutes the structural backbone of the biofilm (Khatoon et al. 2018). It is composed primarily of polysaccharides, proteins, and eDNA, which collectively provide mechanical stability and mediate cell–cell and cell–surface interactions. Mature *Salmonella* biofilms typically range from approximately 50 to 300 µm in thickness under laboratory conditions, whereas thicker biofilms exceeding 500 µm have been reported under nutrient‐rich or prolonged incubation conditions (Vice et al. 2025). As biofilm thickness increases, nutrient and oxygen diffusion become increasingly restricted, generating pronounced physiological gradients that promote metabolic heterogeneity and localized stress adaptation within the biofilm matrix (Murugesan et al. 2026). Beyond structural support, the EPS matrix establishes a heterogeneous microenvironment that affects nutrient diffusion, waste removal, and localized stress exposure (Koutsoumanis et al. 2024). Variations in EPS composition among isolates can lead to distinct biofilm architectures with differing levels of compactness and permeability (Mirghani et al. 2022).

Cell‐to‐cell communication through quorum sensing (QS) plays a central role in coordinating biofilm development. This regulatory mechanism enables bacterial populations to modulate gene expression in response to cell density, thereby synchronizing matrix production, metabolic activity, and dispersal processes (M. Li, Zhao, et al. 2026). QS signals are integrated into broader regulatory networks that respond to environmental cues, such as nutrient availability and stress conditions, ensuring adaptive biofilm behavior (Coolahan and Whalen 2025). At the systems level, biofilm formation is regulated by complex networks involving global transcriptional regulators and stress‐response pathways. These networks dynamically adjust gene expression to optimize survival under fluctuating environmental conditions. Notably, the regulation of biofilm formation is highly context‐dependent, reflecting the interplay between genetic potential and environmental signals (Mitra and Mukhopadhyay 2023).

A critical yet underexplored aspect of *Salmonella* biofilms is the strain‐dependent variation in biofilm architecture. Different isolates can produce biofilms that vary in thickness, density, and spatial organization, which in turn influence their resistance to antimicrobial agents and environmental stresses (D. Wang, Wang, Liu, et al. 2025). For example, confocal laser scanning microscopy (CLSM) has demonstrated substantial isolate‐dependent variation in biofilm thickness and structural compactness, with dense biofilms exhibiting markedly lower antimicrobial penetration than thinner, loosely organized biofilms (Pan et al. 2022). Additionally, biofilms exhibit functional heterogeneity, with subpopulations of cells displaying distinct metabolic states and physiological roles. Persister cells generally represent less than 1% of the total biofilm population under normal conditions but may become substantially enhanced following repeated environmental or antimicrobial stress, thereby contributing disproportionately to treatment failure and long‐term persistence (Kunnath et al. 2024). This heterogeneity contributes to overall biofilm resilience, particularly through the presence of highly tolerant subpopulations (Blanco et al. 2026). Despite advances in molecular characterization, a significant gap remains in establishing clear structure–function relationships within biofilms. Specifically, the degree to which quantitative differences in EPS composition, biofilm thickness, and structural organization determine antimicrobial penetration, stress tolerance, and persistence under industrial processing conditions remains poorly defined. Addressing this gap will be critical for the rational design of targeted antibiofilm strategies (A. K. Singh, Gautam, et al. 2026). Collectively, these mechanisms demonstrate that biofilm formation in Salmonella is governed by tightly coordinated structural and regulatory processes, in which EPS composition, QS, and environmental responsiveness collectively determine biofilm architecture, functional heterogeneity, and persistence potential. The sequential progression from initial surface attachment through EPS‐driven matrix maturation to QS‐coordinated community regulation, together with the strain‐dependent architectural variation that governs antimicrobial penetration and persister cell enrichment in mature biofilms, is schematically illustrated in Figure 3.

**FIGURE 3:**
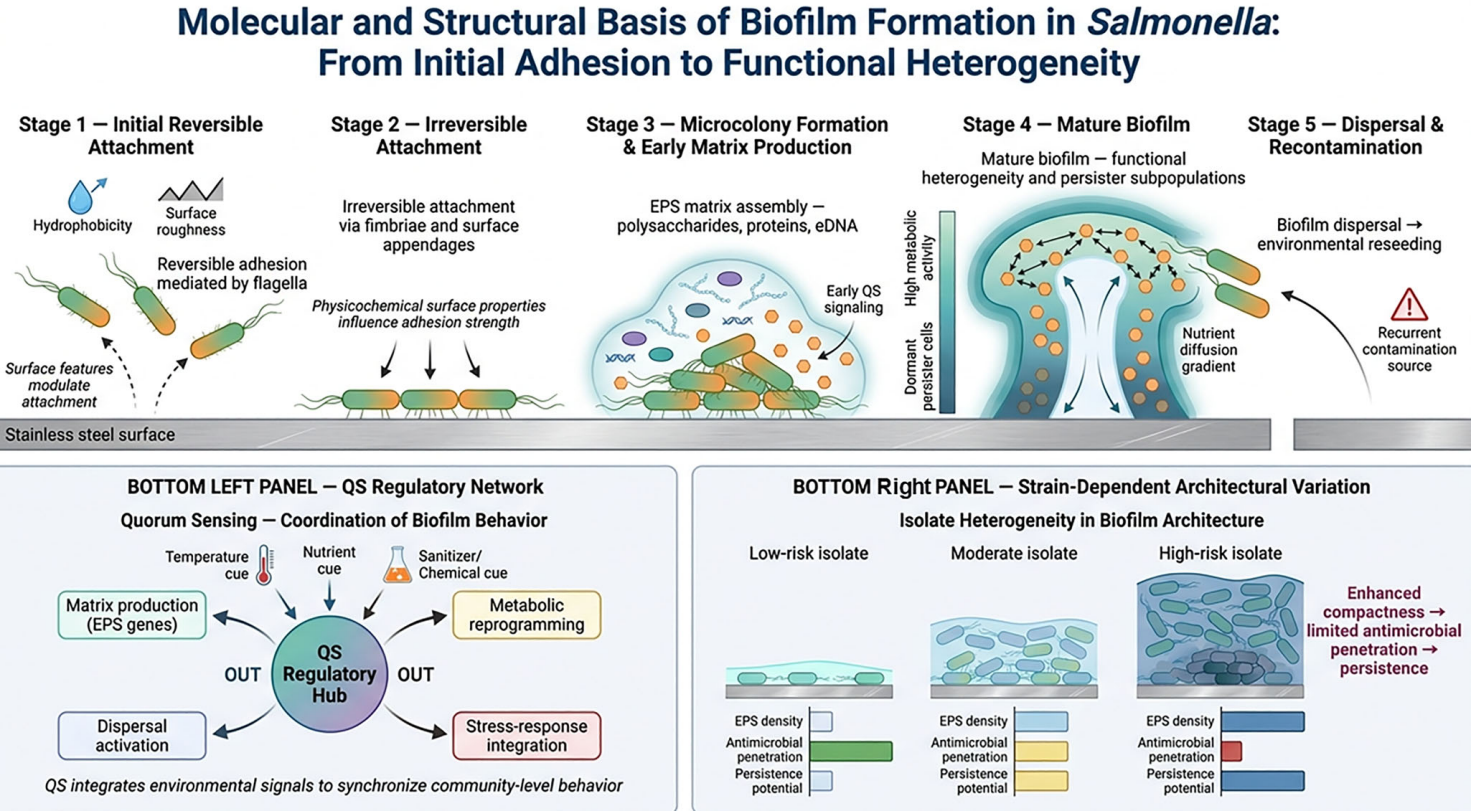
Molecular and structural mechanisms of Salmonella biofilm development and persistence. The upper panel depicts five developmental stages: flagella‐mediated reversible adhesion, fimbriae‐anchored irreversible attachment, microcolony formation with EPS assembly, mature biofilm establishment, and dispersal‐driven recontamination. The EPS matrix (polysaccharides, proteins, eDNA) provides structural stability, establishes nutrient diffusion gradients, and enriches persister cells in deeper layers. The lower‐left panel shows the quorum sensing (QS) network integrating environmental signals (temperature, nutrient limitation, sanitizer exposure) to coordinate matrix production and dispersal. The lower‐right panel demonstrates that increased EPS density and structural compactness in high‐risk isolates progressively reduce antimicrobial penetration and elevate persistence potential. EPS, extracellular polymeric substance; eDNA, extracellular DNA; QS, quorum sensing. Overall, the figure illustrates the coordinated structural and regulatory mechanisms that govern biofilm development, maturation, and persistence in Salmonella.

## 7 Biofilm‐Associated AMR

Building on the genotypic determinants discussed in Section 5 and the molecular and structural mechanisms outlined in Section 6, biofilm‐associated AMR represents a distinct phenotypic and physiological dimension of antimicrobial susceptibility in *Salmonella*. This altered susceptibility results from the combined effects of physical barriers, physiological adaptations, and genetic exchange mechanisms operating within the biofilm microenvironment (Aleksandrowicz et al. 2023). Importantly, biofilm‐associated antimicrobial tolerance should not be considered synonymous with genetically determined AMR. Whereas AMR generally refers to heritable mechanisms that reduce susceptibility to specific antimicrobial agents, biofilm‐associated tolerance may arise from transient physiological states, restricted antimicrobial penetration, metabolic heterogeneity, and stress adaptation without requiring a stable genetic resistance determinant (Uruén et al. 2021). A primary determinant is diffusion limitation, where the extracellular matrix impedes the penetration of antimicrobial agents, resulting in sublethal concentrations reaching deeper biofilm layers (Lu et al. 2026). This gradient reduces bactericidal efficacy and promotes the survival of embedded cells. In parallel, efflux pump systems actively expel antimicrobial compounds from bacterial cells, further decreasing intracellular drug accumulation and contributing to reduced susceptibility (Gaurav et al. 2023). Biofilms also harbor persister cells, a subpopulation characterized by transient metabolic inactivity. These cells are not genetically resistant but exhibit high tolerance to antimicrobial agents due to their dormant state, enabling survival during treatment and subsequent regrowth once selective pressure is removed (Uruén et al. 2021). Additionally, the dense and structured nature of biofilms facilitates HGT, including plasmid exchange, thereby accelerating the dissemination of AMR determinants within and between populations (Michaelis and Grohmann 2023). Beyond these biofilm‐specific mechanisms, phenotypic AMR has been widely reported among *Salmonella* isolates associated with poultry production and poultry‐derived foods (Dlamini et al. 2024). Resistance has been described across multiple antimicrobial classes, including β‐lactams, tetracyclines, aminoglycosides, fluoroquinolones, phenicols, sulfonamides, and polymyxins. However, the distribution of resistance phenotypes varies according to serovar, isolate, geographical region, production stage, and antimicrobial exposure history (Sohail et al. 2026). Representative antimicrobial classes, specific agents, reported *Salmonella* serovars or strains, and associated resistance phenotypes are summarized in Table 2. This evidence demonstrates that AMR in poultry‐associated *Salmonella* is not restricted to a single serovar or antimicrobial class but represents a heterogeneous and multifactorial phenomenon.

**TABLE 2:** Reported antimicrobial resistance and biofilm or sanitizer‐associated tolerance among Salmonella relevant to poultry production and processing environments.

Antimicrobial class	Representative antimicrobial(s)	Reported *Salmonella* serovar or strain	Reported resistance or tolerance phenotype	Poultry‐related context	References
β‐Lactams	Ampicillin, amoxicillin	*S*. Enteritidis strains Z0719SL0002, Z0719SL0007, Z0719SL0011, Z0719SL0012, Z0719SL0013, Z0719SL0014, Z0719SL0018; *S*. Typhimurium	Ampicillin resistance was widespread; all listed *S*. Enteritidis strains showed ampicillin resistance. blaCTX‐M‐15 and blaTEM‐1B were detected in resistant isolates.	Chicken organs, retail chicken meat, bedding, and poultry‐associated environments in Korea	La et al. 2021
Third‐generation cephalosporins	Cefotaxime, ceftiofur, ceftriaxone	*S*. Enteritidis and other poultry‐associated *Salmonella* isolates; representative MDR strains Z0719SL0002–Z0719SL0018	Resistance to cefotaxime, ceftriaxone, and ceftiofur was reported in poultry‐associated isolates; bla CTX‐M‐15 was detected in MDR *S. Enteritidis* strains.	Chicken meat, internal organs, bedding, and slaughterhouse‐associated samples	La et al. 2021; Yang et al. 2024
Fluoroquinolones and quinolones	Ciprofloxacin, nalidixic acid, enrofloxacin	*S*. Enteritidis, *S*. Typhimurium, *S*. Infantis, and other poultry‐associated isolates	Nalidixic acid resistance was common in poultry‐associated isolates; reduced susceptibility to ciprofloxacin was also reported. In Korean retail chicken isolates, gyrA D87Y was frequently associated with ciprofloxacin‐intermediate isolates.	Poultry farms and retail chicken meat	Ali et al. 2025
Tetracyclines	Tetracycline	*S*. Enteritidis, *S. Typhimurium*, *S*. Infantis; representative MDR *S*. Enteritidis strains Z0719SL0002–Z0719SL0018	High levels of tetracycline resistance were reported; tet(A) was detected among MDR poultry‐associated *S*. Enteritidis isolates.	Poultry farms, chicken meat, and poultry‐associated environments	Gargano et al. 2021
Aminoglycosides	Gentamicin, streptomycin, kanamycin	*S*. Typhimurium DT193, DT107, and *S*. Enteritidis; MDR *S*. Enteritidis strains	Streptomycin and gentamicin resistance was reported, including multidrug‐resistant phenotypes containing strA, strB, aph, and aac‐associated determinants.	Commercial poultry farms and chicken‐associated isolates	Punchihewage‐Don et al. 2022
Phenicols	Chloramphenicol, florfenicol	*S*. Enteritidis, *S*. Typhimurium, *S*. Infantis	Chloramphenicol and florfenicol resistance was reported among poultry‐associated isolates; high chloramphenicol resistance was observed among recent Korean retail chicken *Salmonella* isolates.	Retail chicken meat and poultry production chain	C. Wang et al. 2024
Sulfonamides and folate pathway inhibitors	Sulfamethoxazole, trimethoprim, and sulfamethoxazole	*S*. Enteritidis, *S*. Typhimurium, *S*. Infantis	Sulfonamide and trimethoprim‐sulfamethoxazole resistance was reported, with sul2 detected in MDR poultry‐associated *S*. Enteritidis strains.	Poultry farms and chicken meat	La et al. 2021
Polymyxins	Colistin	MDR *S*. Enteritidis strains Z0719SL0002–Z0719SL0018; *S*. Infantis and other poultry‐associated *Salmonella*	Colistin resistance was reported among poultry‐associated MDR isolates; resistance occurred in combination with β‐lactam, tetracycline, and aminoglycoside resistance.	Chicken meat, poultry organs, and associated environments	Di Taranto et al. 2025
Quaternary ammonium compounds (QACs)	Benzalkonium chloride; QAC sanitizers	*S*. Enteritidis, *S*. Kentucky, *S*. Liverpool, *S*. Schwarzengrund and other processing‐environment isolates	Increased tolerance or reduced susceptibility to QACs was observed among isolates recovered from poultry‐processing equipment; responses varied among serovars. Adaptive survival of planktonic and biofilm‐associated *S*. Enteritidis following benzalkonium chloride exposure has also been reported.	Commercial poultry‐processing equipment and sanitation environments	Obe et al. 2021
Chlorine‐based sanitizers	Chlorine, sodium hypochlorite, acidified hypochlorite	*S*. Kentucky, *S*. Liverpool, and other poultry‐processing isolates	Reduced sanitizer susceptibility or tolerance was reported, particularly among biofilm‐associated cells. Biofilms showed greater resistance to disinfectant treatment than planktonic cells.	Poultry‐processing equipment and simulated processing conditions	D. Wang, Fletcher, et al. 2023
Peroxyacetic or peracetic acid	Peracetic acid (PAA)	Environmental *Salmonella* isolates from poultry processing	Biofilm‐associated cells showed greater tolerance than planktonic cells under poultry‐processing conditions; sanitizer efficacy depended on concentration and exposure conditions.	Poultry‐processing environment	Chapagain et al. 2026
Cetylpyridinium chloride (CPC)	CPC	Environmental nontyphoidal *Salmonella* isolates	Biofilm‐associated cells demonstrated increased tolerance relative to planktonic cells, although CPC was effective against most tested biofilms under simulated processing conditions.	Poultry‐processing environment	Rhouma et al. 2020

*Note*: aac, aminoglycoside acetyltransferase gene; aph, aminoglycoside phosphotransferase gene; blaCTX‐M‐15 and blaTEM‐1B, β‐lactamase‐encoding genes; gyrA, DNA gyrase subunit A gene; MDR, multidrug‐resistant; strA and strB, streptomycin resistance genes; sul2, sulfonamide resistance gene; tet(A), tetracycline resistance gene.

Community‐level interactions can further shape AMR in *Salmonella* biofilms, as interactions with resident microbiota may enhance biofilm stability, stress tolerance, and the persistence and dissemination of resistance determinants (Musa et al. 2024; Krüger et al. 2025). Importantly, AMR within biofilms is not uniform but varies across isolates. Isolate‐specific AMR profiles reflect differences in genetic background, regulatory pathways, and prior environmental exposure (Rapacka‐Zdonczyk 2026). Certain isolates exhibit enhanced capacity to maintain resistance traits under biofilm conditions, contributing disproportionately to persistence in processing environments. Furthermore, repeated exposure to sublethal sanitizer concentrations and other processing‐associated stresses can select for or maintain populations with increased sanitizer tolerance and may coincide with enhanced biofilm formation and multidrug‐resistant (MDR) phenotypes, potentially reducing sanitation efficacy if control measures are not properly optimized (Uruén et al. 2021; Krüger et al. 2025; Chapagain et al. 2026). Nevertheless, evidence for a direct causal relationship between sanitizer tolerance, enhanced biofilm formation, and genetically encoded AMR remains heterogeneous, and these phenotypes should therefore be interpreted as interconnected but mechanistically distinct traits (Campillo et al. 2026). This relationship is exemplified by poultry‐associated *Salmonella* Infantis isolates that combine strong biofilm‐forming capacity with MDR characteristics (Musa et al. 2024). Biofilm‐associated tolerance is also particularly relevant to poultry‐processing sanitation, where *Salmonella* may be repeatedly exposed to chlorine‐based compounds, QACs, PAA, and other antimicrobial interventions (Obe et al. 2024). Biofilm‐associated cells can exhibit substantially greater survival than planktonic cells under such conditions because of reduced antimicrobial penetration, altered metabolic activity, and protection by the extracellular matrix (Yaki 2026). Thus, conventional antimicrobial susceptibility profiles alone may not fully predict the survival of *Salmonella* populations embedded in mature biofilms (Edmundo Sitoe et al. 2027). To contextualize these differences, Table 3 presents a comparative framework between planktonic and biofilm‐associated cells, highlighting the key physiological and functional distinctions underlying resistance and persistence. Together, Tables 2 and 3 provide complementary perspectives: Table 3 summarizes the mechanistic differences between planktonic and biofilm‐associated populations, whereas Table 2 provides evidence‐based examples of AMR and tolerance reported among poultry‐associated Salmonella isolates. Collectively, these findings demonstrate that biofilm‐associated AMR reflects the interaction of genetic determinants with biofilm‐specific physiological, structural, and community‐level mechanisms, resulting in enhanced tolerance, persistence, and adaptive resilience in poultry‐processing environments. Accordingly, effective control should not rely solely on conventional antimicrobial susceptibility testing but should also consider biofilm‐associated tolerance, environmental stress adaptation, sanitizer exposure history, and microbial community interactions when evaluating persistence and sanitation efficacy.

**TABLE 3:** Comparative phenotypic and functional characteristics of planktonic versus biofilm‐associated Salmonella cells relevant to antimicrobial resistance, stress adaptation, and persistence in poultry‐processing environments.

Feature	Planktonic cells	Biofilm‐associated cells	References
Cellular organization	Free‐floating	Surface‐attached communities	Bennett et al. 2023
Antimicrobial exposure	Uniform	Concentration gradients	Edison and Kariyawasam 2026
Susceptibility level	High	Reduced	Chaves et al. 2024
EPS matrix	Absent	Present (protective barrier)	Aleksandrowicz et al. 2023
Antimicrobial penetration	Unrestricted	Limited diffusion	Aleksandrowicz et al. 2023
Metabolic activity	Uniform, active	Heterogeneous (active + dormant)	Guillín et al. 2025
Efflux pump activity	Baseline	Upregulated	Aleksandrowicz et al. 2023
Persister cell population	Rare	Enhanced	Z. Wang, Wang, Dong, et al. 2025
Stress tolerance	Moderate	High	Jia et al. 2017
Horizontal gene transfer	Low frequency	Enhanced frequency	Linn et al. 2026
Gene expression	Stable	Dynamic, stress‐responsive	Hassanin et al. 2025
Resistance development	Slower	Accelerated under stress	Almatroudi 2025
Recovery after treatment	Limited	Rapid regrowth possible	Joos et al. 2025
Adaptation to environment	Limited	Highly adaptive	Cadena et al. 2019
Role in contamination	Transient	Persistent reservoir	Arslan et al. 2026

Note: The phenotypic characteristics summarized in this table represent generalized trends observed for planktonic and biofilm‐associated Salmonella populations. In commercial poultry‐processing environments, these phenotypes may be further influenced by synergistic interactions with resident microbiota and mixed‐species biofilm communities, which can modify biofilm architecture, stress tolerance, antimicrobial susceptibility, and persistence.

Abbreviations: AMR, antimicrobial resistance; EPS, extracellular polymeric substances.

## 8 Poultry‐Processing‐Induced Stress Adaptation and Cross‐Protection

Processing environments impose dynamic and frequently sublethal stresses that reshape the physiology of *Salmonella*, with direct consequences for persistence and control. Rather than acting independently, these stresses collectively select for phenotypes better adapted to surviving cleaning cycles and fluctuating conditions characteristic of poultry‐processing lines (Marcu et al. 2026a). Repeated exposure to these stresses promotes physiological plasticity, enabling Salmonella to rapidly adjust cellular metabolism, membrane composition, and stress‐response pathways that collectively enhance environmental fitness. Such adaptive responses contribute to the long‐term establishment of persistent populations within equipment surfaces, drains, conveyor systems, and other difficult‐to‐sanitize niches commonly encountered in poultry‐processing facilities (Avcı et al. 2026).

### 8.1 Processing Stresses

Key stressors include thermal variation (heat during scalding and transient cold during chilling), desiccation on intermittently wet surfaces, and sublethal exposure to sanitizers arising from dilution, organic load, or insufficient contact time (Koutsoumanis et al. 2024). These conditions rarely achieve complete lethality; instead, they impose selective pressures that enrich for stress‐tolerant subpopulations. Notably, the frequency and sequence of these exposures can influence the adaptive trajectory, favoring isolates capable of rapid physiological adjustment (Rapacka‐Zdonczyk 2026). In commercial poultry‐processing plants, these stressors rarely occur in isolation but instead act sequentially or simultaneously throughout successive processing operations. Consequently, Salmonella populations are repeatedly challenged by fluctuating temperature, nutrient limitation, oxidative stress, osmotic stress, and chemical sanitizers, creating complex selective environments that favor highly adaptable phenotypes capable of surviving multiple intervention steps (Pradhan and Devi Negi 2019).

### 8.2 Adaptive Mechanisms

Adaptive responses are orchestrated through global gene regulation, enabling rapid reprogramming of cellular functions in response to environmental cues. These responses include increased production of extracellular matrix components, which enhances structural robustness and metabolic reconfiguration toward energy‐efficient states that support survival under nutrient limitation and stress (Xue et al. 2026). Collectively, these adaptations improve tolerance to subsequent antimicrobial interventions and facilitate stable surface colonization. Stress adaptation is coordinated through interconnected regulatory networks involving alternative sigma factors, two‐component regulatory systems, and global transcriptional regulators that collectively modulate membrane integrity, oxidative stress defense, osmoprotection, and biofilm‐associated gene expression (Gaca and Lemos 2019). Activation of these regulatory pathways promotes EPS production, curli fimbriae synthesis, and cellulose biosynthesis, thereby strengthening biofilm architecture while reducing susceptibility to environmental and antimicrobial stresses (Castelijn et al. 2012).

A defining feature of these responses is cross‐protection, whereby exposure to one stress confers increased tolerance to other, unrelated stresses (Izydorcyzk and Church 2026). For example, prior exposure to thermal or osmotic stress can enhance resistance to chemical disinfectants. This phenomenon reflects overlapping regulatory pathways and shared protective mechanisms, resulting in broad‐spectrum resilience that complicates control strategies (Lu et al. 2026). Sublethal exposure to sanitizers may also induce adaptive responses that reduce subsequent susceptibility to oxidative disinfectants and other antimicrobial interventions. Although these responses do not necessarily confer stable genetic resistance, repeated exposure can transiently increase stress tolerance, thereby reducing sanitation efficacy and facilitating persistence under industrial conditions (Zhou et al. 2026).

A central outcome of stress adaptation is the reinforcement of biofilm‐associated traits, linking environmental exposure to persistence and antimicrobial tolerance. In this context, stress not only selects for survival but also actively promotes a transition toward more resilient community states (Almatroudi 2025). Conceptually, this relationship can be described as a stress adaptation–biofilm–AMR axis, in which repeated exposure to sublethal stress drives regulatory responses that strengthen biofilm structure and elevate resistance phenotypes (Elshobary et al. 2025). Despite its significance, integration of stress adaptation with biofilm dynamics and AMR remains insufficiently resolved under actual processing conditions (Coppola et al. 2025). Importantly, biofilm‐associated cells experience spatially heterogeneous stress conditions that generate phenotypic diversity within the same microbial community. This heterogeneity enables subsets of cells to survive antimicrobial exposure and subsequently recolonize processing surfaces, contributing to recurrent contamination events despite routine sanitation procedures (Azeem et al. 2025). Future studies should integrate transcriptomic, proteomic, metabolomic, and environmental surveillance approaches to better characterize stress adaptation under commercial processing conditions. Such knowledge will facilitate the development of intervention strategies that target regulatory pathways governing adaptive responses rather than relying solely on increasing sanitizer concentration or application frequency (Mekonnen 2026).

Elucidating these interactions is critical for designing interventions that disrupt adaptive pathways rather than inadvertently reinforcing them (V. Singh, Sinha, et al. 2026). The role of processing‐induced stress adaptation in driving biofilm reinforcement and AMR development in Salmonella is illustrated in Figure 4. Overall, repeated exposure to sublethal processing stresses drives coordinated regulatory responses that couple biofilm reinforcement with AMR in *Salmonella*, thereby enhancing persistence under industrial conditions.

**FIGURE 4:**
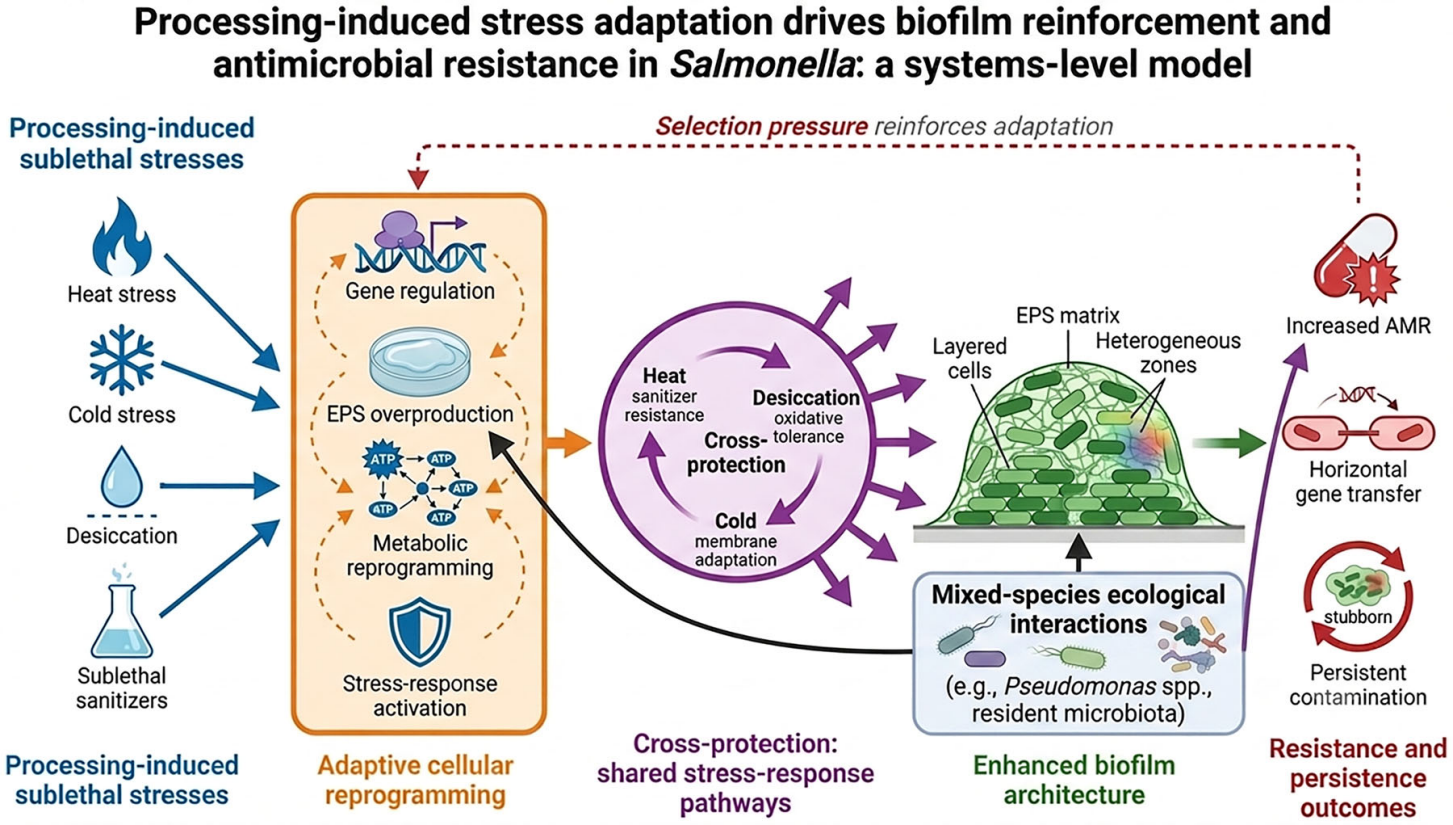
Processing‐induced stress adaptation promotes biofilm reinforcement and antimicrobial resistance in Salmonella. Sublethal stresses encountered during poultry processing trigger adaptive cellular responses, including global gene regulation, extracellular polymeric substance (EPS) overproduction, and metabolic reprogramming, which converge via cross‐protection mechanisms to enable resistance to multiple stressors. Within mixed‐species biofilms, ecological interactions with resident microorganisms further influence EPS organization, nutrient utilization, stress adaptation, and antimicrobial tolerance, resulting in more structured and resilient biofilm communities than those formed by Salmonella alone. The resulting enhancement of biofilm architecture increases structural stability and antimicrobial tolerance, facilitating persistence under processing conditions. These community‐level interactions may also promote the maintenance and dissemination of antimicrobial resistance determinants, reinforcing long‐term environmental persistence and recurrent contamination. Feedback between adaptive responses, mixed‐species community dynamics, and resistance mechanisms further strengthens this cycle during repeated environmental exposure. Overall, the figure emphasizes that persistent Salmonella contamination results from the combined effects of isolate‐specific stress adaptation and community‐level ecological interactions within mixed‐species biofilms.

## 9 Detection, Surveillance, and Control Challenges

Accurate detection and quantification of *Salmonella* in poultry‐processing environments remain constrained by methodological limitations, particularly for biofilm‐associated populations. Current approaches rely primarily on culture‐based assays and molecular techniques, both of which exhibit reduced reliability under conditions reflective of industrial systems (Armstrong et al. 2025). Culture‐based methods, although widely accepted for regulatory purposes, are inherently limited in their ability to recover cells embedded within biofilms. Strong surface attachment and matrix protection hinder cell detachment, leading to systematic underestimation of contamination levels. Additionally, enrichment steps can introduce bias by favoring fast‐growing populations, thereby obscuring the presence of more persistent or stress‐adapted subpopulations (Vo et al. 2026).

Molecular detection methods, including PCR‐based assays, offer enhanced sensitivity and specificity but lack the ability to discriminate between viable and nonviable cells. This limitation is particularly relevant following sanitation procedures, where residual DNA from inactivated cells may lead to overestimation of contamination. Furthermore, these methods are typically optimized for planktonic samples and require additional sample processing to disrupt biofilms, which can compromise reproducibility (H. Chen et al. 2026). An additional complication arises from the presence of viable but nonculturable (VBNC) cells, which are frequently induced under processing‐related stress conditions. These cells evade detection by conventional culture methods yet retain the capacity to resuscitate and contribute to subsequent contamination (Arfan and Zhao 2026). As a result, standard detection workflows may fail to capture the full spectrum of microbial states present in processing environments. Collectively, these constraints reveal a central gap in current monitoring strategies: the difficulty of accurately detecting, quantifying, and characterizing biofilm‐associated and stress‐adapted *Salmonella* populations under realistic processing conditions (Maroju et al. 2026). Addressing this gap will require the development and validation of biofilm‐targeted, viability‐informed, and potentially real‐time detection approaches capable of distinguishing viable populations and resolving spatial heterogeneity within complex industrial environments. Importantly, improved detection should not be viewed solely as an analytical objective; it is also fundamental to identifying persistent contamination reservoirs and informing risk‐based control strategies (Valde et al. 2026).

The implications for control are particularly important because the contamination risk associated with *Salmonella* cannot be adequately assessed solely from its presence or absence. Biofilm‐forming capacity varies considerably among serovars and among isolates within the same serovar, indicating that isolate‐level characteristics should be considered when evaluating persistence potential and prioritizing intervention strategies (M. Kim et al. 2024). Similarly, although surface properties can influence bacterial attachment and biofilm development, no food‐contact material can be considered universally resistant to *Salmonella* colonization. Material selection should therefore complement, rather than replace, effective cleaning and sanitation programs (Elbehiry and Alajaji 2026). The persistence of *S*. Minnesota and other serovars capable of maintaining biofilm‐associated survival under refrigeration further illustrates the limitations of relying on temperature control alone. Although chilling remains essential for limiting bacterial growth, low temperatures may not completely prevent biofilm establishment or environmental persistence. Consequently, control measures targeting attachment, biofilm development, and survival during chilling and subsequent processing stages should be considered alongside conventional temperature management (de Melo et al. 2021).

Furthermore, studies evaluating sanitizer efficacy have demonstrated that the protective advantage conferred by biofilms is strongly influenced by the presence of organic matter. As a result, disinfectant performance assessed under clean laboratory conditions may not accurately reflect effectiveness in commercial processing environments (Koti et al. 2024). Sanitation efficacy should therefore be evaluated under experimentally relevant soiling conditions and against biofilm‐associated populations that more closely represent those encountered in industrial settings. In addition, emerging food safety technologies that integrate real‐time monitoring, data‐driven decision‐making, and targeted biofilm‐control strategies may further enhance sanitation effectiveness in complex processing environments (Reem et al. 2025). Taken together, these considerations support a shift from generalized *Salmonella* control toward risk‐based and evidence‐driven strategies that integrate detection, ecological surveillance, isolate‐level characterization, and targeted intervention. Such an approach should prioritize persistent environmental reservoirs and high‐persistence phenotypes rather than treating all detected *Salmonella* populations as epidemiologically equivalent. Integrating advanced detection technologies with realistic sanitation assessment and targeted biofilm‐control interventions may ultimately improve the identification and elimination of persistent contamination sources throughout the poultry‐processing continuum (Edison and Kariyawasam 2026).

## 10 Translational Control Strategies

Controlling *Salmonella* in poultry‐processing environments remains a persistent challenge, primarily due to the disconnect between laboratory efficacy and industrial performance (Gentile et al. 2025). Although numerous antimicrobial interventions have been developed, their effectiveness is often diminished under real processing conditions, where biofilm‐associated resilience and environmental variability limit consistent outcomes. Bridging this gap requires a translational perspective that rigorously assesses both mechanistic efficacy and practical feasibility (Akter et al. 2026). A successful translational approach should integrate microbiological efficacy with engineering feasibility, economic sustainability, regulatory acceptance, and compatibility with existing processing workflows. Such considerations are essential for ensuring that promising laboratory interventions can be reliably implemented on a commercial scale (Alqahtani and Altoaimi 2026; H. Kim et al. 2026).

### 10.1 Limitations of Conventional Sanitizers

Conventional sanitizers, including chlorine‐based compounds, PAA, and QACs, remain the primary tools for microbial control in poultry processing (Ciluveru et al. 2026). However, their performance against biofilm‐associated populations is frequently suboptimal. Limited penetration through the extracellular matrix reduces effective antimicrobial exposure, while adaptive physiological states further decrease susceptibility (Coenye 2023). Additionally, operational constraints such as high organic load, insufficient contact time, and uneven surface coverage significantly reduce treatment efficiency. These limitations contribute to incomplete biofilm removal and facilitate the persistence of contamination hotspots (Mitra et al. 2026). Repeated reliance on conventional sanitizers may also impose selective pressures that favor stress‐adapted or biofilm‐associated populations, particularly when sanitizers are applied below recommended concentrations or under inadequate cleaning conditions (Jiang and Gänzle 2026). Consequently, sanitation effectiveness depends not only on disinfectant selection but also on optimization of cleaning protocols, equipment design, biofilm removal efficiency, and process verification through environmental monitoring (Makovska et al. 2025).

### 10.2 Emerging Antibiofilm Strategies

In response to these challenges, advanced antibiofilm strategies have been developed to target specific structural and functional aspects of biofilms (H. Li, Fan, et al. 2026). Natural antimicrobials offer multitarget activity and reduced chemical residues, although their stability and consistency under processing conditions require further validation (A. Kumar, Das, et al. 2025). Enzymatic treatments are promising for disrupting matrix integrity and enhancing the accessibility and efficacy of co‐applied antimicrobials (Murugaiyan et al. 2022). Bacteriophage‐based interventions offer high specificity and the potential for self‐propagation within susceptible populations, but their effectiveness can be influenced by strain variability and environmental factors (Muneeb et al. 2025). Ultraviolet light‐emitting diode (UV‐LED) technologies provide rapid, nonchemical surface decontamination, but their application is limited by surface geometry and penetration depth. Similarly, antiadhesive and functionalized surfaces aim to prevent initial microbial attachment, representing a preventive strategy with long‐term potential (Rudhart et al. 2020). Increasing evidence suggests that no single intervention is sufficient to eliminate mature biofilms under industrial conditions. Consequently, hurdle‐based approaches that combine physical, chemical, and biological interventions have attracted considerable attention because they simultaneously target extracellular matrix disruption, microbial inactivation, and inhibition of recolonization (Mishra et al. 2023). Such integrated strategies may improve treatment efficacy while reducing dependence on high concentrations of conventional chemical sanitizers. Furthermore, advances in smart sanitation technologies, including sensor‐assisted environmental monitoring, automated cleaning systems, and real‐time microbial detection, have the potential to improve intervention timing and optimize sanitation performance within poultry‐processing facilities (Chowdhury et al. 2025; Chowdhury et al. 2023).

Despite their promise, the transition of these strategies from laboratory to industrial application remains limited. Key challenges include scalability across complex processing systems, cost implications of implementation, and regulatory compliance (Gujjar et al. 2025). Moreover, the effectiveness of these interventions is highly context‐dependent, influenced by surface properties, processing conditions, and microbial composition. Critically, efficacy demonstrated under controlled laboratory conditions does not necessarily translate to reliable performance in industrial environments (Su et al. 2025). This discrepancy underscores the need for integrated approaches that combine multiple interventions targeting both biofilm structure and microbial physiology while aligning with operational and regulatory constraints (Metris et al. 2025).

Another important limitation is that many experimental studies evaluate interventions using single‐species laboratory biofilms formed on idealized surfaces, which inadequately represent the multispecies communities, aged biofilms, and complex food residues encountered in commercial poultry‐processing plants (Arthur et al. 2024). Future validation should therefore incorporate pilot‐scale and industrial‐scale studies using realistic processing conditions and naturally contaminated equipment to better predict practical performance (L. Liu et al. 2026). Successful industrial implementation will also require standardized evaluation protocols, cost–benefit analyses, regulatory approval pathways, and integration with existing food safety management systems such as HACCP and environmental monitoring programs (Radu et al. 2023). Collaboration among researchers, equipment manufacturers, regulatory agencies, and the poultry industry will be essential for accelerating the adoption of innovative antibiofilm technologies (Rugarabamu and Gaspary Mwanyika 2025).

Future progress will depend on the development of robust, scalable, and economically viable strategies validated under realistic processing conditions, ensuring practical applicability for improving food safety outcomes. Bridging the gap between laboratory efficacy and industrial performance is essential to deliver reliable, scalable interventions against Salmonella in processing environments.

## 11 Translational Gaps and Future Research Roadmap

Building on the translational control strategies discussed above, several important knowledge gaps remain that limit their successful implementation under commercial poultry‐processing conditions. Advancing the control of Salmonella in poultry‐processing environments requires addressing several critical knowledge gaps that continue to limit the translation of scientific discoveries into effective industrial interventions. Despite substantial progress in understanding biofilm formation, stress adaptation, and AMR, significant uncertainties remain regarding how these mechanisms interact under commercial processing conditions. Most current studies rely on simplified laboratory models that fail to capture the complexity of industrial environments, including heterogeneous food‐contact surfaces, fluctuating environmental stresses, mixed‐species microbial communities, and variable organic loads (Ugwu et al. 2025). Consequently, the predictive value and industrial scalability of many proposed control strategies remain uncertain. A major conceptual gap is the limited integration of isolate heterogeneity, stress adaptation, and AMR within unified experimental frameworks. These determinants are frequently investigated independently despite increasing evidence that persistence emerges from their dynamic interaction (H. Kim et al. 2026). As a result, the mechanisms underlying the development of high‐risk persistent phenotypes remain incompletely understood, limiting the ability to predict contamination recurrence and intervention outcomes (Huemer et al. 2020). Furthermore, multiomics datasets integrating genomics, transcriptomics, proteomics, and metabolomics remain scarce for processing‐relevant Salmonella isolates, restricting efforts to identify regulatory networks that govern persistence under industrial conditions (Sanches et al. 2024).

Addressing these challenges requires a coordinated research strategy spanning short‐, medium‐, and long‐term priorities. In the short term (1–3 years), research should prioritize standardized characterization of high‐risk Salmonella isolates and serotypes of concern (SOCs) associated with poultry production, integrating phenotypic traits such as biofilm‐forming capacity, stress tolerance, persistence, AMR, and virulence potential rather than considering virulence alone. Such characterization will facilitate more accurate risk stratification and support the development of targeted intervention strategies for serotypes with the greatest public health and industrial significance (Katz et al. 2024). Research should also focus on the development of processing‐relevant biofilm models and the generation of integrated multiomics datasets linked to well‐defined phenotypes. Particular emphasis should be placed on establishing reproducible experimental frameworks that enable comparison across studies and facilitate identification of persistence‐associated biomarkers (Subramanian et al. 2020). Medium‐term priorities (3–7 years) should focus on integrating multiomics datasets with predictive modeling approaches capable of linking genotype, phenotype, and environmental conditions. Combining mechanistic models with machine‐learning algorithms may improve prediction of biofilm development, stress adaptation trajectories, contamination persistence, and intervention efficacy across diverse processing scenarios (Rubrice et al. 2025). Validation of these models under pilot‐scale and industrial conditions will be essential for improving their practical applicability. Long‐term priorities (7–10 years) should aim to establish fully integrated precision‐control systems capable of real‐time contamination monitoring and adaptive intervention. Advances in biosensing technologies, digital twins, artificial intelligence (AI), and automated sanitation platforms have the potential to transform conventional food safety management into predictive and responsive systems. Such technologies could enable continuous environmental surveillance, early detection of high‐risk contamination events, and dynamic optimization of sanitation protocols based on real‐time risk assessment (Gheibi et al. 2026). A critical requirement across all time horizons is the generation of industrial‐scale validation data. Future studies should move beyond proof‐of‐concept laboratory investigations and prioritize collaborations among academia, industry, technology developers, and regulatory agencies. Such multidisciplinary approaches will be essential for translating mechanistic insights into scalable interventions capable of reducing persistent contamination under commercial conditions.

Figure 5 illustrates a translational roadmap linking multiomics integration, predictive modeling, and smart sanitation technologies to next‐generation control strategies for *Salmonella* in poultry‐processing environments. Collectively, future progress will depend on the transition from descriptive investigations toward integrated, systems‐level frameworks capable of predicting persistence, identifying high‐risk isolates, and enabling precision‐based intervention strategies. Bridging these knowledge gaps will be essential for achieving sustainable and scalable control of biofilm‐mediated *Salmonella* contamination in modern poultry‐processing systems.

**FIGURE 5:**
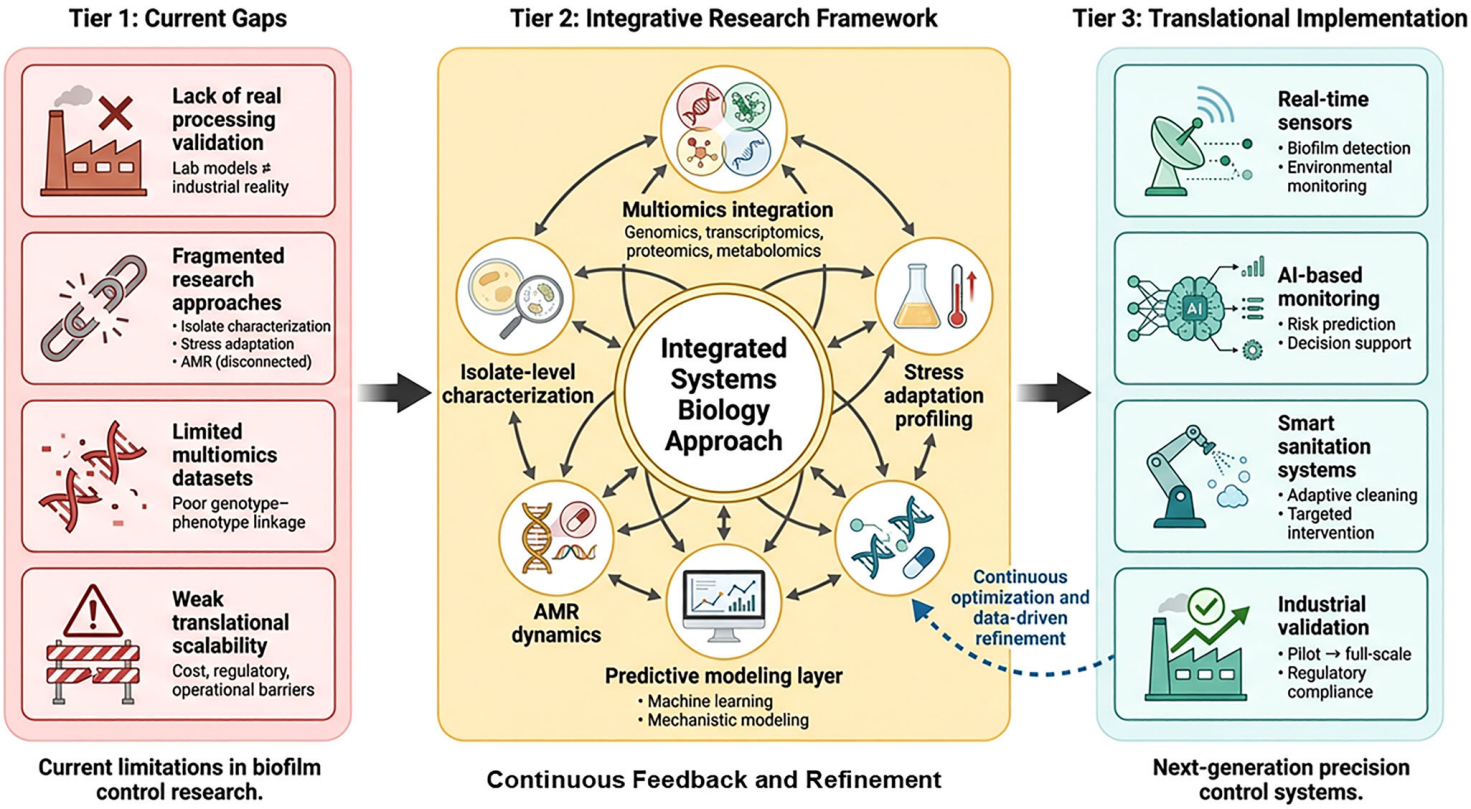
Translational roadmap for next‐generation control of persistent Salmonella in poultry‐processing environments. The figure outlines current limitations, such as lack of industrial validation, fragmented research approaches, and limited multiomics data. Integration of isolate‐level characterization, stress adaptation, and antimicrobial resistance (AMR) within a multiomics framework enables predictive modeling of biofilm behavior and resistance dynamics. These insights support the development of smart sanitation systems incorporating real‐time sensing and artificial intelligence (AI)‐based monitoring. Continuous feedback between industrial implementation and research refinement is essential for achieving scalable, data‐driven, precision‐based control strategies. Overall, the figure presents a translational framework linking fundamental research, predictive multiomics approaches, industrial implementation, and continuous feedback to support precision‐based control of persistent Salmonella in poultry‐processing environments.

## 12 Longitudinal Investigations of Persistent Contamination

Several longitudinal investigations have provided direct evidence that Salmonella persistence in poultry‐processing facilities results from the long‐term establishment of resident populations rather than repeated introduction of unrelated strains. These investigations provide valuable insights into the ecological and operational factors that sustain contamination despite routine sanitation (Marin et al. 2022; Merino et al. 2019). Longitudinal environmental investigations have repeatedly demonstrated the recovery of genetically related *Salmonella* strains from the same processing facilities over extended periods, confirming the existence of resident contamination sources capable of surviving conventional control measures (Siceloff et al. 2025). For example, repeated isolation of genetically indistinguishable Salmonella strains from drains, conveyor systems, and equipment surfaces over multiple production cycles has demonstrated that processing facilities can harbor stable environmental reservoirs capable of repeatedly contaminating finished products (Q. Chen et al. 2024). These findings highlight the importance of understanding contamination as a dynamic ecological process rather than a series of isolated introduction events. A common theme emerging from contamination investigations is the identification of previously unrecognized environmental reservoirs within processing operations. Equipment design complexity, inaccessible structural components, moisture‐retaining sites, and production‐associated workflow patterns have frequently been implicated in sustaining contamination despite routine sanitation (Koovarjee et al. 2026).

Several investigations further demonstrated that structural deficiencies, including hollow rollers, damaged conveyor belts, inaccessible welds, and drainage systems, provided protected niches that limited sanitizer penetration and promoted long‐term biofilm persistence (Ban‐Cucerzan et al. 2025). Such observations underscore the importance of environmental mapping and source‐tracking approaches for identifying contamination reservoirs that may remain undetected by conventional monitoring programs. Contamination investigations have also revealed that successful control often depends on addressing operational and infrastructural factors rather than relying solely on intensified sanitation. Improvements in hygienic equipment design, environmental surveillance programs, zoning practices, and source‐tracking strategies have frequently produced more sustainable outcomes than chemical interventions alone (Shi et al. 2026). In several documented investigations, elimination of persistent contamination was achieved only after facility modifications, equipment redesign, or replacement of chronically contaminated components, illustrating that sanitation alone was insufficient to eradicate established environmental reservoirs (Akdemir Evrendilek 2026). Increasing application of whole‐genome sequencing (WGS) has further strengthened contamination investigations by enabling differentiation between sporadic contamination events and long‐term resident populations (Brown et al. 2021). WGS has also enabled confirmation of transmission pathways linking environmental isolates with product contamination, thereby supporting evidence‐based corrective actions and more targeted environmental monitoring programs (Sophian et al. 2026).

Collectively, these longitudinal investigations demonstrate that effective management of persistent *Salmonella* contamination requires an integrated understanding of facility ecology, environmental reservoirs, and contamination pathways. Importantly, these investigations consistently show that persistent contamination arises from interactions among microbial adaptation, biofilm formation, facility design, and operational practices rather than from any single contributing factor. The lessons derived from these investigations provide a practical foundation for developing more targeted and sustainable control strategies within poultry‐processing systems.

## 13 Regulatory Frameworks and Policy Implications

Current regulatory frameworks for *Salmonella* control in poultry processing, including those established by USDA Food Safety and Inspection Service (FSIS), the European Union (EU), and Codex Alimentarius, have substantially improved food safety through microbiological performance standards, process‐control verification, and preventive management systems. The USDA‐FSIS regulatory approach emphasizes pathogen reduction performance standards, verification sampling, and the implementation of preventive control measures within poultry establishments (USDA‐FSIS 2021); the European regulatory framework, supported by EFSA risk assessments, focuses on farm‐to‐fork control strategies, epidemiological surveillance, reduction of Salmonella prevalence throughout the production chain, and, overall, the One Health strategy (EFSA and ECDC 2025). However, most existing regulations remain primarily focused on pathogen occurrence and product compliance, with limited consideration of factors that contribute to contamination persistence within processing environments (Betiku et al. 2025).

A major challenge for current regulatory systems is the translation of isolate‐level findings, such as enhanced biofilm‐forming capacity, stress tolerance, and AMR profiles, into actionable food safety policies. Although these characteristics provide valuable mechanistic insights into pathogen persistence, regulatory frameworks generally lack standardized criteria for incorporating microbial phenotypic traits into routine risk assessment and control decision‐making (Yu et al. 2025). Recent advances in molecular epidemiology and environmental surveillance have created opportunities to strengthen regulatory approaches by incorporating risk‐based assessment of contamination sources and transmission pathways. Greater emphasis on environmental monitoring, source attribution, and longitudinal surveillance could improve the identification of recurring contamination problems and support more effective corrective actions (Majumdar et al. 2026). Integrating biofilm‐associated persistence and AMR‐related characteristics into risk‐based regulatory models represents an important future direction. Such approaches could enable the identification of high‐risk processing niches, improve targeted sanitation strategies, and complement existing microbiological criteria by addressing the environmental reservoirs responsible for recurrent contamination events (Ugwu et al. 2025). From a policy perspective, future regulatory strategies should increasingly emphasize proactive prevention of Salmonella establishment, persistence, and cross‐contamination within poultry‐processing environments, rather than relying primarily on end‐product compliance testing (Marcu et al. 2026b). Integrating environmental data, molecular source‐tracking tools, and risk‐based decision frameworks into food safety management systems may improve the long‐term effectiveness of *Salmonella* control programs while supporting continuous improvement across poultry‐processing operations (Kuppusamy et al. 2024). Such preventive approaches are intended to reduce contamination risks throughout processing and should not be interpreted as implying the complete elimination of Salmonella or a zero‐tolerance objective (Betiku et al. 2025).

As scientific understanding of contamination ecology continues to evolve, regulatory policies will need to adapt accordingly. Future regulatory development should therefore establish stronger links between microbial ecology research and practical control measures, enabling evidence‐based integration of emerging concepts such as biofilm persistence, stress adaptation, and resistance dissemination into poultry safety management systems. Strengthening the connection between emerging scientific evidence and regulatory decision‐making will be essential for achieving more sustainable and proactive control of *Salmonella* in modern poultry‐processing systems.

## 14 Limitations of This Review

Although this review provides a comprehensive synthesis of current knowledge regarding biofilm‐mediated Salmonella persistence in poultry‐processing environments, several limitations should be considered. The available literature remains highly heterogeneous with respect to experimental design, biofilm models, environmental conditions, and analytical methodologies, which complicates direct comparisons among studies and may influence the interpretation of reported findings. In addition, much of the current understanding is derived from laboratory‐based investigations that may not fully reflect the complexity of commercial processing environments. The limited availability of longitudinal studies, industrial‐scale validation, and integrated datasets linking isolate heterogeneity, stress adaptation, and AMR further constrains the development of predictive frameworks for persistence. Moreover, differences in sampling strategies, experimental conditions, and definitions of persistence across studies may limit the generalizability of individual findings. These limitations highlight important gaps in the current evidence base and should be considered when interpreting the mechanisms and control strategies discussed in this review. Future research addressing these gaps through standardized methodologies, longitudinal monitoring, and industrial‐scale validation will be essential for improving risk assessment, intervention design, and industrial implementation.

## 15 Conclusion

Effective control of *Salmonella* in poultry‐processing environments requires a shift from generalized approaches toward integrated, risk‐based strategies that consider pathogen diversity, environmental complexity, and processing conditions. Although isolate‐level characterization provides valuable insights into specific virulence, biofilm‐forming, and resistance‐associated traits, persistence within poultry‐processing environments is fundamentally a community‐level phenomenon shaped by interactions among diverse microbial populations, resident microbiota, environmental reservoirs, and repeated selective pressures. Recognizing strain heterogeneity and ecological interactions is therefore crucial for identifying high‐risk contamination scenarios and developing more effective control measures. Future interventions should move beyond targeting individual isolates and instead address the broader ecological processes that support survival, adaptation, and dissemination of *Salmonella* within processing systems. Progress will depend on integrated strategies that concurrently consider biofilm architecture, adaptive stress responses, AMR mechanisms, sanitation performance, and microbial community dynamics rather than treating these factors independently. These strategies must be tailored to real processing constraints to ensure consistent, practical, and scalable outcomes. The integration of predictive tools, environmental monitoring, and microbial characterization approaches may enhance risk assessment and decision‐making; however, precision biomarker‐based strategies should be considered complementary tools rather than standalone solutions until their predictive reliability and applicability across diverse processing environments are further validated (Koutsoumanis et al. 2024). Advancing food safety outcomes will require stronger translational research efforts that bridge mechanistic discoveries with industrial validation and regulatory implementation. Ultimately, sustainable *Salmonella* control will depend on systems‐level approaches that integrate microbial ecology, technological innovation, and evidence‐based management practices to reduce pathogen persistence and improve contamination control in modern poultry‐processing systems.

## Nomenclature

AI: artificial intelligence
AMR: antimicrobial resistance
CLSM: confocal laser scanning microscopy
eDNA: extracellular DNA
EPS: extracellular polymeric substance
ESBL: extended‐spectrum β‐lactamase
EU: European Union
HGT: horizontal gene transfer
MDR: multidrug‐resistant
OD: optical density
PAA: peracetic acid
QACs: quaternary ammonium compounds
QS: quorum sensing
FSIS: Food Safety and Inspection Service
UV‐LED: ultraviolet light‐emitting diode
VBNC: viable but nonculturable
WGS: whole‐genome sequencing

## Author Contributions

**Md Anamul Hasan Chowdhury**: methodology, software, visualization, writing – review and editing, writing – original draft, conceptualization. **Chowdhury Sanat Anjum Reem**: conceptualization, methodology, software, visualization, writing – original draft, writing – review and editing. **Md Furkanur Rahaman Mizan**: conceptualization, validation. **Md. Ashrafudoulla**: conceptualization, validation. **Sang‐Do Ha**: conceptualization, funding acquisition, investigation, supervision.

## Conflicts of Interest

The authors declare no conflicts of interest.

## Data Availability

No new data were created or analyzed in this study. Data sharing is not applicable to this article.

## References

[crf370674-bib-0001] Abrego, A. , B. Wiseman , and S. E. Gragg . 2025. “Summarizing the Current Knowledge and Existing Knowledge Gaps for Preharvest and Postharvest *Salmonella* Contamination in Pork.” Journal of Food Protection 88, no. 10: 100609. 10.1016/j.jfp.2025.100609. 40876777

[crf370674-bib-0002] Adel, O. , M. F. Khallaf , and H. M. Elhariry . 2025. “Molecular Identification and Disinfectant Susceptibility of Biofilm‐Forming Bacteria in Meat and Poultry Processing Establishments.” Arab Universities Journal of Agricultural Sciences 33, no. 1: 66–80. 10.21608/ajs.2025.364329.1598.

[crf370674-bib-0003] Adeyemi, O. D. , and S. N. Nahashon . 2026. “Mitigating *Salmonella* in Poultry Using Probiotics: Mechanisms, Challenges, and Opportunities.” Microorganisms 14, no. 2: 365. 10.3390/microorganisms14020365. PMC12942985 41753652

[crf370674-bib-0004] Akdemir Evrendilek, G. 2026. “The Hygiene Continuum in Seafood Processing: Integrating Design, Sanitation, and Workforce Safety for Sustainable Food Systems.” Hygiene 6, no. 1: 6. 10.3390/hygiene6010006.

[crf370674-bib-0005] Akter, S. , M. A. Rahman , M. Ashrafudoulla , et al. 2026. “Targeting Biofilm Resistance in Meat Production: Postbiotics as a Clean‐Label Alternative, a Bibliometric Analysis Review.” Poultry Science 105, no. 3: 106362. 10.1016/j.psj.2025.106362. PMC12805358 41485351

[crf370674-bib-0006] Albaseer, S. S. , G. E. Morlock , B. Ringbeck , L. Böhm , and H. Hollert . 2026. “Atmospheric Pesticide Drift and the Integrity of Organic Supply Chains: Pathways, Risks, and Regulatory Gaps.” Journal of Hazardous Materials Advances 23: 101263. 10.1016/j.hazadv.2026.101263.

[crf370674-bib-0007] Aleksandrowicz, A. , E. Carolak , A. Dutkiewicz , A. Błachut , W. Waszczuk , and K. Grzymajlo . 2023. “Better Together–*Salmonella* Biofilm‐Associated Antibiotic Resistance.” Gut Microbes 15, no. 1: 2229937. 10.1080/19490976.2023.2229937. PMC10321201 37401756

[crf370674-bib-0008] Ali, M. S. , H.‐S. Kang , B.‐Y. Moon , et al. 2025. “Prevalence and Characterization of Ciprofloxacin‐Resistant *Salmonella enterica* spp. Isolated From Food Animals During 2010–2023 in South Korea.” Veterinary Quarterly 45, no. 1: 1–11. 10.1080/01652176.2025.2473733. PMC11915734 40091866

[crf370674-bib-0009] Almatroudi, A. 2025. “Biofilm Resilience: Molecular Mechanisms Driving Antibiotic Resistance in Clinical Contexts.” Biology 14, no. 2: 165. 10.3390/biology14020165. PMC11852148 40001933

[crf370674-bib-0010] Alqahtani, A. , and B. Altoaimi . 2026. “Innovative Advances in Microalgal High‐Value Products: Unlocking Potential From Bioprocessing to Bioindustrial Applications.” Frontiers in Bioengineering and Biotechnology 14: 1737889. 10.3389/fbioe.2026.1737889. PMC13062254 41969533

[crf370674-bib-0011] Arfan, H. M. , and X. Zhao . 2026. “Graduate Student Literature Review: Viable but Nonculturable State in *Staphylococcus aureus*—A Potential Implication for Food Safety in the Context of Bovine Mastitis.” Journal of Dairy Science 109, no. 6: 6468–6477. 10.3168/jds.2025-27810. 41850359

[crf370674-bib-0012] Arksey, H. , and L. O'Malley . 2005. “Scoping Studies: Towards a Methodological Framework.” International Journal of Social Research Methodology 8, no. 1: 19–32. 10.1080/1364557032000119616.

[crf370674-bib-0013] Armstrong, C. M. , C.‐Y. Chen , Y. Xie , et al. 2025. “Quantification of *Salmonella* in Raw Poultry Using Droplet Digital PCR With a Whole Cell, Enrichment‐Free Approach.” Journal of Food Protection 88, no. 5: 100498. 10.1016/j.jfp.2025.100498. 40139502

[crf370674-bib-0014] Arslan, S. , R. Schena , A. Romano , M. Hohenegger , L. De Martino , and F. P. Nocera . 2026. “Characterization of Antimicrobial Resistance and Biofilm Formation in *Escherichia coli* Isolated From Wild Boars.” Research in Veterinary Science 204: 106148. 10.1016/j.rvsc.2026.106148. 41864121

[crf370674-bib-0015] Arthur, M. , E. L. Afari , E.‐A. Alexa , et al. 2024. “Recent Advances in Examining the Factors Influencing the Efficacy of Biocides Against *Listeria monocytogenes* Biofilms in the Food Industry: A Systematic Review.” Comprehensive Reviews in Food Science and Food Safety 24, no. 1: e70083. 10.1111/1541-4337.70083. PMC11684510 39736097

[crf370674-bib-0016] Avcı, F. N. , N. Akçelik , and M. Akçelik . 2026. “Resilient Communities: The Role of Biofilms in *Salmonella enterica* Ecology, Persistence and Pathogenesis.” Environmental Microbiology 28, no. 2: e70250. 10.1111/1462-2920.70250. 41656623

[crf370674-bib-0017] Azeem, K. , S. Fatima , A. Ali , A. Ubaid , F. M. Husain , and M. Abid . 2025. “Biochemistry of Bacterial Biofilm: Insights Into Antibiotic Resistance Mechanisms and Therapeutic Intervention.” Life 15, no. 1: 49. 10.3390/life15010049. PMC11767195 39859989

[crf370674-bib-0018] Ban‐Cucerzan, A. , K. Imre , A. Morar , et al. 2025. “Persistent Threats: A Comprehensive Review of Biofilm Formation, Control, and Economic Implications in Food Processing Environments.” Microorganisms 13, no. 8: 1805. 10.3390/microorganisms13081805. PMC12388178 40871309

[crf370674-bib-0019] Barbieri, L. 2020. “Biocide‐Releasing and Nanostructured Surfaces: Fabrication, Characterisation and Bacterial Response.” Doctoral thesis, The University of Liverpool. https://livrepository.liverpool.ac.uk/3119371/1/201153714_Nov2020.pdf.

[crf370674-bib-0020] Bashir, A. , A. Azeem , Y. Stedman , and A. C. Hilton . 2019. “Pet Food Factory Isolates of *Salmonella* Serotypes Do Not Demonstrate Enhanced Biofilm Formation Compared to Serotype‐Matched Clinical and Veterinary Isolates.” BioMed Research International 2019: 8569459. 10.1155/2019/8569459. PMC6374821 30834278

[crf370674-bib-0021] Bennett, A. N. , K. J. Woolard , A. Sorge , C. Melander , and J. S. Gunn . 2023. “Spectrum of Activity of *Salmonella* Anti‐Biofilm Compounds: Evaluation of Activity Against Biofilm‐Forming ESKAPE Pathogens.” Biofilm 6: 100158. 10.1016/j.bioflm.2023.100158. PMC10542598 37790732

[crf370674-bib-0022] Betiku, E. , T. T. Ogundipe , T. Kalapala , and T. Obe . 2025. “A Mini‐Review on Multi‐Hurdle Control of *Salmonella* Along Poultry Production Continuum.” Animals 15, no. 6: 875. 10.3390/ani15060875. PMC11939138 40150404

[crf370674-bib-0023] Bezek, K. , J. Avberšek , O. Zorman Rojs , and D. Barlič‐Maganja . 2023. “Antimicrobial and Antibiofilm Effect of Commonly Used Disinfectants on *Salmonella* Infantis Isolates.” Microorganisms 11, no. 2: 301. 10.3390/microorganisms11020301. PMC9958858 36838265

[crf370674-bib-0024] Białucha, A. , E. Gospodarek‐Komkowska , J. Kwiecińska‐Piróg , and K. Skowron . 2020. “Influence of Selected Factors on Biofilm Formation by *Salmonella enterica* Strains.” Microorganisms 9, no. 1: 43. 10.3390/microorganisms9010043. PMC7824446 33375734

[crf370674-bib-0025] Blanco, K. C. , P. de Figueiredo , J. A. Willis , et al. 2026. “Layer‐Dependent Modulation of *S. aureus* Biofilms by Light‐Induced Oxidative Stress.” Current Research in Microbial Sciences 10: 100571. 10.1016/j.crmicr.2026.100571. PMC12968421 41810130

[crf370674-bib-0026] Blaznik, M. , M. Volk , B. Kraigher , et al. 2025. “Biofilm Structure as a Key Factor in Antibiotic Tolerance: Insights From *Bacillus subtilis* Model Systems.” npj Biofilms and Microbiomes 11, no. 1: 232. 10.1038/s41522-025-00864-x. PMC12714748 41413428

[crf370674-bib-0027] Brasão, S. C. , R. T. Melo , R. R. Prado , et al. 2021. “Characterization and Control of Biofilms of *Salmonella* Minnesota of Poultry Origin.” Food Bioscience 39: 100811. 10.1016/j.fbio.2020.100811.

[crf370674-bib-0028] Brown, E. W. , R. Bell , G. Zhang , et al. 2021. “ *Salmonella* Genomics in Public Health and Food Safety.” EcoSal Plus 9, no. 2: eESP00082020. 10.1128/ecosalplus.esp-0008-2020. PMC11163839 34125583

[crf370674-bib-0029] Bundurus, I. A. , I. Balta , I. Pet , et al. 2024. “Mechanistic Concepts Involved in Biofilm Associated Processes of *Campylobacter jejuni*: Persistence and Inhibition in Poultry Environments.” Poultry Science 103, no. 12: 104328. 10.1016/j.psj.2024.104328. PMC11483643 39366290

[crf370674-bib-0030] Cadena, M. , T. Kelman , M. L. Marco , and M. Pitesky . 2019. “Understanding Antimicrobial Resistance (AMR) Profiles of *Salmonella* Biofilm and Planktonic Bacteria Challenged With Disinfectants Commonly Used During Poultry Processing.” Foods 8, no. 7: 275. 10.3390/foods8070275. PMC6678331 31336660

[crf370674-bib-0031] Campillo, R. , I. García‐Penas , D. Berdejo , D. García‐Gonzalo , and R. Pagán . 2026. “Antimicrobial Resistance as a Potential Gateway to Cross‐Protection Against Food‐Related Stressors in *Salmonella enterica* .” Journal of Agriculture and Food Research 29: 103114. 10.1016/j.jafr.2026.103114.

[crf370674-bib-0032] Castelijn, G. A. A. , J. A. Parabirsing , M. H. Zwietering , R. Moezelaar , and T. Abee . 2013. “Surface Behaviour of *S*. Typhimurium, *S*. Derby, *S*. Brandenburg and *S*. Infantis.” Veterinary Microbiology 161, no. 3–4: 305–314. 10.1016/j.vetmic.2012.07.047. 22906529

[crf370674-bib-0033] Castelijn, G. A. A. , S. van der Veen , M. H. Zwietering , R. Moezelaar , and T. Abee . 2012. “Diversity in Biofilm Formation and Production of Curli Fimbriae and Cellulose of *Salmonella* Typhimurium Strains of Different Origin in High and Low Nutrient Medium.” Biofouling 28, no. 1: 51–63. 10.1080/08927014.2011.648927. 22235813

[crf370674-bib-0034] Castello, A. , C. Massaro , E. Seghers , et al. 2025. “Isolation and Molecular Characterization of Antimicrobial‐Resistant Bacteria From Vegetable Foods.” Pathogens 14, no. 7: 682. 10.3390/pathogens14070682. PMC12300653 40732728

[crf370674-bib-0035] Chandross‐Cohen, T. , T. Chung , S. C. Watson , M. L. Rolon , and J. Kovac . 2025. “Precision Food Safety: Advances in Omics‐Based Surveillance for Proactive Detection and Management of Foodborne Pathogens.” Trends in Food Science & Technology 163: 105186. 10.1016/j.tifs.2025.105186.

[crf370674-bib-0036] Chapagain, S. , R. Y. Otwey , U. Ghimire , B. Adhikari , and J. Dhakal . 2026. “Application of Peracetic Acid in Poultry Processing: Effects of Treatment Dynamics and Emerging Risk of Resistance Development in *Salmonella* spp.” Comprehensive Reviews in Food Science and Food Safety 25, no. 2: e70445. 10.1111/1541-4337.70445. PMC12988585 41830500

[crf370674-bib-0037] Chard, A. N. , T. Eisenstein , A. Cote , and S. Kremer‐Caldwell . 2025. “ *Salmonella* Illness Outbreaks Attributed to Chicken by Product Type, United States, 1998–2022.” Journal of Food Protection 88, no. 12: 100660. 10.1016/j.jfp.2025.100660. 41161595

[crf370674-bib-0038] Chaves, R. D. , S. H. Kumazawa , A. M. Khaneghah , V. O. Alvarenga , H. M. Hungaro , and A. S. Sant'Ana . 2024. “Comparing the Susceptibility to Sanitizers, Biofilm‐Forming Ability, and Biofilm Resistance to Quaternary Ammonium and Chlorine Dioxide of 43 *Salmonella enterica* and *Listeria monocytogenes* Strains.” Food Microbiology 117: 104380. 10.1016/j.fm.2023.104380. 37918997

[crf370674-bib-0039] Chawla, H. , P. Anand , K. Garg , et al. 2023. “A Comprehensive Review of Microbial Contamination in the Indoor Environment: Sources, Sampling, Health Risks, and Mitigation Strategies.” Frontiers in Public Health 11: 1285393. 10.3389/fpubh.2023.1285393. PMC10701447 38074709

[crf370674-bib-0040] Chen, H. , J. Xiao , B. Diao , S. Zheng , H. Shao , and L. Liu . 2026. “Recent Advances in Bacterial Detection Techniques Targeting Genetic, Protein, and Enzymatic Biomarkers.” *Journal of Pharmaceutical Analysis*. ahead of print, February 9. 10.1016/j.jpha.2026.101584.

[crf370674-bib-0041] Chen, Q. , V. Palanisamy , R. Wang , J. M. Bosilevac , and S. Chitlapilly Dass . 2024. “ *Salmonella*‐Induced Microbiome Profile in Response to Sanitation by Quaternary Ammonium Chloride.” Microbiology Spectrum 12, no. 2: e0234623. 10.1128/spectrum.02346-23. PMC10846233 38226804

[crf370674-bib-0042] Chen, S. H. , N. Fegan , C. Kocharunchitt , J. P. Bowman , and L. L. Duffy . 2020. “Impact of Poultry Processing Operating Parameters on Bacterial Transmission and Persistence on Chicken Carcasses and Their Shelf Life.” Applied and Environmental Microbiology 86, no. 12: e00594. 10.1128/aem.00594-20. PMC7267199 32276979

[crf370674-bib-0043] Cheng, G. , J. Ning , S. Ahmed , et al. 2019. “Selection and Dissemination of Antimicrobial Resistance in Agri‐Food Production.” Antimicrobial Resistance & Infection Control 8, no. 1: 158. 10.1186/s13756-019-0623-2. PMC6805589 31649815

[crf370674-bib-0044] Cheng, R. A. , C. R. Eade , and M. Wiedmann . 2019. “Embracing Diversity: Differences in Virulence Mechanisms, Disease Severity, and Host Adaptations Contribute to the Success of Nontyphoidal *Salmonella* as a Foodborne Pathogen.” Frontiers in Microbiology 10: 1368. 10.3389/fmicb.2019.01368. PMC6611429 31316476

[crf370674-bib-0045] Chowdhury, M. A. H. , M. Ashrafudoulla , S. I. U. Mevo , M. F. R. Mizan , S. H. Park , and S. D. Ha . 2023. “Current and Future Interventions for Improving Poultry Health and Poultry Food Safety and Security: A Comprehensive Review.” Comprehensive Reviews in Food Science and Food Safety 22, no. 3: 1555–1596. 10.1111/1541-4337.13121. 36815737

[crf370674-bib-0046] Chowdhury, M. A. H. , C. S. A. Reem , M. Ashrafudoulla , et al. 2025. “Role of Advanced Cleaning and Sanitation Techniques in Biofilm Prevention on Dairy Equipment.” Comprehensive Reviews in Food Science and Food Safety 24, no. 3: e70176. 10.1111/1541-4337.70176. 40260792

[crf370674-bib-0047] Ciluveru, V. , J. Simon , J. M. Farber , S. D. Ryan , C. R. Kothapalli , and D. S. Munther . 2026. “Peracetic Acid Efficacy and Decay Kinetics in Poultry Processing Under Chiller Conditions.” International Journal of Food Microbiology 448: 111572. 10.1016/j.ijfoodmicro.2025.111572. 41389788

[crf370674-bib-0048] Coenye, T. 2023. “Biofilm Antimicrobial Susceptibility Testing: Where Are We and Where Could We Be Going?” Clinical Microbiology Reviews 36, no. 4: e0002423. 10.1128/cmr.00024-23. PMC10732061 37812003

[crf370674-bib-0049] Coolahan, M. , and K. E. Whalen . 2025. “A Review of Quorum‐Sensing and Its Role in Mediating Interkingdom Interactions in the Ocean.” Communications Biology 8, no. 1: 179. 10.1038/s42003-025-07608-9. PMC11794697 39905218

[crf370674-bib-0050] Coppola, F. , F. Fratianni , V. Bianco , et al. 2025. “New Methodologies as Opportunities in the Study of Bacterial Biofilms, Including Food‐Related Applications.” Microorganisms 13, no. 5: 1062. 10.3390/microorganisms13051062. PMC12114119 40431235

[crf370674-bib-0051] Counihan, K. L. , S. Tilman , J. Uknalis , S. Mukhopadhyay , B. A. Niemira , and D. Bermudez‐Aguirre . 2025. “Attachment and Biofilm Formation of Eight Different *Salmonella* Serotypes on Three Food‐Contact Surfaces at Different Temperatures.” Microorganisms 13, no. 7: 1446. 10.3390/microorganisms13071446. PMC12298123 40731956

[crf370674-bib-0053] Desmousseaux, C. , M. Guilbaud , G. Jard , et al. 2025. “Biofilm Formation in Dairy: A Food Safety Concern—Biofilms in the Milking Machine, From Laboratory Scale to On‐Farm Results.” Journal of Dairy Science 108, no. 8: 8120–8140. 10.3168/jds.2024-25352. 40049401

[crf370674-bib-0054] Di Taranto, P. , F. Petruzzi , G. Normanno , et al. 2025. “Prevalence and Antimicrobial Resistance of *Salmonella* Strains Isolated From Chicken Samples in Southern Italy.” Microorganisms 13, no. 2: 270. 10.3390/microorganisms13020270. PMC11858494 40005636

[crf370674-bib-0055] Dlamini, S. B. , V. Mlambo , C. M. Mnisi , and C. N. Ateba . 2024. “Virulence, Multiple Drug Resistance, and Biofilm‐Formation in *Salmonella* Species Isolated From Layer, Broiler, and Dual‐Purpose Indigenous Chickens.” PLoS ONE 19, no. 10: e0310010. 10.1371/journal.pone.0310010. PMC11515961 39466757

[crf370674-bib-0056] Drauch, V. , C. Kornschober , N. Palmieri , M. Hess , and C. Hess . 2021. “Infection Dynamics of *Salmonella* Infantis Strains Displaying Different Genetic Backgrounds—With or Without pESI‐Like Plasmid—Vary Considerably.” Emerging Microbes & Infections 10, no. 1: 1471–1480. 10.1080/22221751.2021.1951124. PMC8300933 34197273

[crf370674-bib-0057] Edison, L. K. , and S. Kariyawasam . 2026. “Comparative Biofilmomics of Antimicrobial‐Resistant *Salmonella*: Serovar‐ and Host‐Specific Signatures.” Animals 16, no. 9: 1302. 10.3390/ani16091302. PMC13163011 42121722

[crf370674-bib-0058] Edmundo Sitoe, E. P. , M. P. Silva Láscaris , P. Monteiro Evangelista , M. S. Alves dos Santos , M. A. Moshood , and A. S. Sant'Ana . 2027. “Integrated Predictive Modeling and Machine Learning Analysis of *Salmonella enterica* Serovars Senftenberg and Typhimurium Survival in Black Pepper Under Different Treatment Conditions and Storage Temperatures.” Food Control 191: 112470. 10.1016/j.foodcont.2026.112470.

[crf370674-bib-0102] EFSA Panel on Biological Hazards (BIOHAZ), K. Koutsoumanis, A. Allende, et al .. 2022. “Transmission of Antimicrobial Resistance (AMR) During Animal Transport.” EFSA Journal 20, no. 10: e07586. 10.2903/j.efsa.2022.7586. PMC9593722 36304831

[crf370674-bib-0103] EFSA Panel on Biological Hazards (BIOHAZ), K. Koutsoumanis, A. Allende, et al .. 2024. “Persistence of Microbiological Hazards in Food and Feed Production and Processing Environments.” EFSA Journal 22, no. 1: e8521. 10.2903/j.efsa.2024.8521. PMC10797485 38250499

[crf370674-bib-0061] El Kadri, H. , K. M. Costello , P. Thomas , et al. 2021. “The Antimicrobial Efficacy of Remote Cold Atmospheric Plasma Effluent Against Single and Mixed Bacterial Biofilms of Varying Age.” Food Research International 141: 110126. 10.1016/j.foodres.2021.110126. 33641993

[crf370674-bib-0060] Elbehiry, A. , and A. I. Alajaji . 2026. “Next‐Generation Strategies for Controlling Foodborne Pathogens: Precision Antimicrobials, Biofilm Disruption, and Emerging Molecular Interventions.” Foods 15, no. 2: 194. 10.3390/foods15020194. PMC12840017 41596793

[crf370674-bib-0062] Elshobary, M. E. , N. K. Badawy , Y. Ashraf , et al. 2025. “Combating Antibiotic Resistance: Mechanisms, Multidrug‐Resistant Pathogens, and Novel Therapeutic Approaches: An Updated Review.” Pharmaceuticals 18, no. 3: 402. 10.3390/ph18030402. PMC11944582 40143178

[crf370674-bib-0059] European Food Safety Authority (ECDC), and European Centre for Disease Prevention and Control (ECDC) . 2025. “The European Union One Health 2024 Zoonoses Report.” EFSA Journal 23, no. 12: e9759. 10.2903/j.efsa.2025.9759. PMC12686834 41377167

[crf370674-bib-0063] Fadipe, E. O. , and L. E. Hölzle . 2025. “Phylogenetic Analysis and Public Health Implications of *Salmonella* Strains in Southwestern States of Nigeria Using invA Gene Sequences.” Animals 15, no. 23: 3399. 10.3390/ani15233399. PMC12691423 41375458

[crf370674-bib-0064] Featherstone, A. , A. C. Brown , and S. Chitlapilly Dass . 2023. “Understanding How Different Surfaces and Environmental Biofilms Found in Food Processing Plants Affect the Spread of COVID‐19.” PLoS ONE 18, no. 6: e0286659. 10.1371/journal.pone.0286659. PMC10246802 37285373

[crf370674-bib-0073] Gómez, N. C. , J. M. P. Ramiro , B. V. Quecan , and B. D. G. de Melo Franco . 2016. “Use of Potential Probiotic Lactic Acid Bacteria (LAB) Biofilms for the Control of *Listeria monocytogenes*, *Salmonella* Typhimurium and *Escherichia coli* O157:H7 Biofilm Formation.” Frontiers in Microbiology 7: 863. 10.3389/fmicb.2016.00863. PMC4901071 27375584

[crf370674-bib-0066] Gaca, A. O. , and J. A. Lemos . 2019. “Adaptation to Adversity: The Intermingling of Stress Tolerance and Pathogenesis in Enterococci.” Microbiology and Molecular Biology Reviews 83, no. 3: e00008–19. 10.1128/mmbr.00008-19. PMC6710459 31315902

[crf370674-bib-0067] Gargano, V. , S. Sciortino , D. Gambino , et al. 2021. “Antibiotic Susceptibility Profile and Tetracycline Resistance Genes Detection in *Salmonella* spp. Strains Isolated From Animals and Food.” Antibiotics 10, no. 7: 809. 10.3390/antibiotics10070809. PMC8300615 34356729

[crf370674-bib-0068] Gaurav, A. , P. Bakht , M. Saini , S. Pandey , and R. Pathania . 2023. “Role of Bacterial Efflux Pumps in Antibiotic Resistance, Virulence, and Strategies to Discover Novel Efflux Pump Inhibitors.” Microbiology 169, no. 5: 001333. 10.1099/mic.0.001333. PMC10268834 37224055

[crf370674-bib-0069] Gentile, N. , L. Lorenzo‐Rebenaque , A. Marco‐Fuertes , L. Montoro‐Dasi , and C. Marin . 2025. “Emerging Challenges in *Salmonella* Control: The Need for Innovative and Sustainable Disinfection Strategies in Poultry Farming.” Pathogens 14, no. 9: 912. 10.3390/pathogens14090912. PMC12472306 41011812

[crf370674-bib-0070] Georganas, A. , G. Graziosi , E. Catelli , and C. Lupini . 2024. “ *Salmonella enterica* Serovar Infantis in Broiler Chickens: A Systematic Review and Meta‐Analysis.” Animals 14, no. 23: 3453. 10.3390/ani14233453. PMC11640450 39682418

[crf370674-bib-0071] Gharat, Y. P. , A. G. Abdelhamid , and A. E. Yousef . 2025. “Enhancing the Destruction of *Burkholderia cepacia* Biofilm on Stainless Steel Coupons by Combining Matrix‐Degrading Enzymes With Antimicrobials.” Frontiers in Cellular and Infection Microbiology 15: 1662291. 10.3389/fcimb.2025.1662291. PMC12641019 41293055

[crf370674-bib-0072] Gheibi, M. , M. Palušak , M. Salava , et al. 2026. “A Smart Decision Support System for Co^2+^ Preconcentration Using RSM and AI‐Driven Adsorptive Modeling.” Engineering Science and Technology, an International Journal 77: 102335. 10.1016/j.jestch.2026.102335.

[crf370674-bib-0074] Gorski, L. , N. W. Shariat , A. K. Richards , A. T. Siceloff , A. A. Noriega , and D. M. Harhay . 2024. “Growth Assessment of *Salmonella enterica* Multi‐Serovar Populations in Poultry Rinsates With Commonly Used Enrichment and Plating Media.” Food Microbiology 119: 104431. 10.1016/j.fm.2023.104431. 38225041

[crf370674-bib-0076] Gržinić, G. , A. Piotrowicz‐Cieślak , A. Klimkowicz‐Pawlas , et al. 2023. “Intensive Poultry Farming: A Review of the Impact on the Environment and Human Health.” Science of the Total Environment 858: 160014. 10.1016/j.scitotenv.2022.160014. 36368402

[crf370674-bib-0075] Grimont, P. A. , and F. X. Weill . 2007. Antigenic Formulae of the Salmonella Serovars. 9th ed. WHO Collaborating Centre for Reference and Research on Salmonella.

[crf370674-bib-0077] Gu, G. , L. K. Strawn , J. Zheng , E. A. Reed , and S. L. Rideout . 2019. “Diversity and Dynamics of *Salmonella enterica* in Water Sources, Poultry Litters, and Field Soils Amended With Poultry Litter in a Major Agricultural Area of Virginia.” Frontiers in Microbiology 10: 2868. 10.3389/fmicb.2019.02868. PMC6951424 31956319

[crf370674-bib-0078] Guillín, Y. , C. Ortiz , and W. Hidalgo . 2025. “Comparative Metabolic Study of Planktonic and Sessile Cells in *Salmonella* Enteritidis ATCC 13076: Elucidating Metabolic Pathways Driving Biofilm Formation.” PLoS ONE 20, no. 1: e0317420. 10.1371/journal.pone.0317420. PMC11761094 39854347

[crf370674-bib-0079] Gujjar, S. , S. Kukal , P. Jayabal , et al. 2025. “Nanomaterials for Biomedical Applications: Addressing Regulatory Hurdles and Strategic Solutions.” Nano Trends 11: 100127. 10.1016/j.nwnano.2025.100127.

[crf370674-bib-0080] Habib, I. , M.‐Y. I. Mohamed , G. B. Lakshmi , et al. 2025. “First Detection and Genomic Analysis of Mcr‐1‐Positive *Salmonella* Infantis Isolated From a Broiler Production System in the United Arab Emirates.” Frontiers in Veterinary Science 12: 1592955. 10.3389/fvets.2025.1592955. PMC12417193 40933520

[crf370674-bib-0081] Hammons, S. R. 2024. “Response to Questions Posed by the Food Safety and Inspection Service: Enhancing *Salmonella* Control in Poultry Products.” Journal of Food Protection 87, no. 2: 100168. 10.1016/j.jfp.2023.100168. 37939849

[crf370674-bib-0082] Hassanin, A. , B. Lories , and H. P. Steenackers . 2025. “ *Salmonella* Stress Response Systems as Targets for Anti‐Virulence Strategies.” BMC Microbiology 25, no. 1: 378. 10.1186/s12866-025-04003-6. PMC12217294 40596824

[crf370674-bib-0083] He, Y. , J. Zhao , X. Xu , et al. 2026. “Genotypic and Phenotypic Strategies to Detect Antibiotic Resistance in Foodborne Pathogens Along the Food Processing Chain.” Current Research in Food Science 12: 101379. 10.1016/j.crfs.2026.101379. PMC12990374 41847557

[crf370674-bib-0084] Hindieh, P. , J. Yaghi , J. C. Assaf , et al. 2025. “Emerging Multimodal Strategies for Bacterial Biofilm Eradication: A Comprehensive Review.” Microorganisms 13, no. 12: 2796. 10.3390/microorganisms13122796. PMC12735920 41471999

[crf370674-bib-0085] Huemer, M. , S. Mairpady Shambat , S. D. Brugger , and A. S. Zinkernagel . 2020. “Antibiotic Resistance and Persistence—Implications for Human Health and Treatment Perspectives.” The EMBO Reports 21, no. 12: e51034. 10.15252/embr.202051034. PMC7726816 33400359

[crf370674-bib-0086] Hussain, R. , G. Stenhouse , A. Ismail Ahmed , et al. 2025. “Heterogeneous *Salmonella* Typhi Transmission Within a Household: Genomic Insights From a Chronic Carrier.” Journal of Medical Microbiology 74, no. 9: 002070. 10.1099/jmm.0.002070. PMC12476145 40982564

[crf370674-bib-0087] Ivers, C. , E. C. Kaya , U. Yucel , D. Boyle , and V. Trinetta . 2024. “Evaluation of *Salmonella* Biofilm Attachment and Hydrophobicity Characteristics on Food Contact Surfaces.” BMC Microbiology 24, no. 1: 387. 10.1186/s12866-024-03556-2. PMC11447956 39363349

[crf370674-bib-0088] Izydorcyzk, C. , and D. L. Church . 2026. “Evolution and Rise of Antimicrobial Resistance in Key Public Health Bacteria Within and Across the One Health Sectors.” Future Microbiology 21, no. 2: 181–215. 10.1080/17460913.2026.2633088. PMC12987526 41792991

[crf370674-bib-0089] Jia, K. , G. Wang , L. Liang , M. Wang , H. Wang , and X. Xu . 2017. “Preliminary Transcriptome Analysis of Mature Biofilm and Planktonic Cells of *Salmonella* Enteritidis Exposure to Acid Stress.” Frontiers in Microbiology 8: 1861. 10.3389/fmicb.2017.01861. PMC5622974 29018430

[crf370674-bib-0090] Jiang, Y. C. , and M. G. Gänzle . 2026. “Resistance of Multispecies Biofilms to Sanitization With Chlorine, Peroxyacetic Acid, and Ozone Nano‐Bubble−Activated Water.” Journal of Food Protection 89, no. 4: 100719. 10.1016/j.jfp.2026.100719. 41690401

[crf370674-bib-0091] Joos, M. , S. Van Ginneken , X. Villanueva , et al. 2025. “EPS Inhibitor Treatment of *Salmonella* Impacts Evolution Without Selecting for Resistance to Biofilm Inhibition.” npj Biofilms and Microbiomes 11, no. 1: 73. 10.1038/s41522-025-00693-y. PMC12056028 40328762

[crf370674-bib-0092] Justino, L. , A. A. S. Baptista , R. H. de Carvalho , et al. 2026. “Environmental Persistence and Genotypic and Phenotypic Characterization of *Salmonella* Minnesota in Poultry Slaughterhouses.” Pathogens 15, no. 3: 247. 10.3390/pathogens15030247. PMC13029514 41901701

[crf370674-bib-0093] Katz, T. S. , D. M. Harhay , J. W. Schmidt , and T. L. Wheeler . 2024. “Identifying a List of *Salmonella* Serotypes of Concern to Target for Reducing Risk of Salmonellosis.” Frontiers in Microbiology 15: 1307563. 10.3389/fmicb.2024.1307563. PMC10894960 38410382

[crf370674-bib-0094] Khalefa, H. S. , A. Elkelish , M. S. Khattab , et al. 2025. “ *Salmonella* in Broiler Chickens: Biofilm Formation, Disinfectant Resistance and Contribution to Microbial Risk in Housing Environments.” Pakistan Veterinary Journal 45, no. 3: 1179–1189. 10.29261/pakvetj/2025.242.

[crf370674-bib-0095] Khatoon, Z. , C. D. McTiernan , E. J. Suuronen , T.‐F. Mah , and E. I. Alarcon . 2018. “Bacterial Biofilm Formation on Implantable Devices and Approaches to Its Treatment and Prevention.” Heliyon 4, no. 12: e01067. 10.1016/j.heliyon.2018.e01067. PMC6312881 30619958

[crf370674-bib-0096] Kim, H. , M. A. Haque , M. A. Razzak , M. J. Jang , S. Song , and S. Ku . 2026. “Probiotic Development Strategy Centered on Stability and Regulatory Considerations.” Comprehensive Reviews in Food Science and Food Safety 25, no. 1: e70320. 10.1111/1541-4337.70320. PMC12770815 41491675

[crf370674-bib-0097] Kim, M. , C. Barnett‐Neefs , R. A. Chavez , E. Kealey , M. Wiedmann , and M. J. Stasiewicz . 2024. “Risk Assessment Predicts Most of the Salmonellosis Risk in Raw Chicken Parts Is Concentrated in Those Few Products With High Levels of High‐Virulence Serotypes of *Salmonella* .” Journal of Food Protection 87, no. 7: 100304. 10.1016/j.jfp.2024.100304. 38777091

[crf370674-bib-0098] Kim, S.‐H. , S. Jyung , and D.‐H. Kang . 2022. “Comparative Study of *Salmonella* Typhimurium Biofilms and Their Resistance Depending on Cellulose Secretion and Maturation Temperatures.” LWT ‐ Food Science and Technology 154: 112700. 10.1016/j.lwt.2021.112700.

[crf370674-bib-0099] Kim, T. , Y. Kim , H. Kim , et al. 2025. “Prevalence of *Salmonella* Serotypes Isolated From Clinical Samples in Chicken Farms and Meat in Slaughterhouses in South Korea.” Poultry Science 104, no. 6: 105147. 10.1016/j.psj.2025.105147. PMC12018171 40228339

[crf370674-bib-0100] Koovarjee, B. , M. Becker , J. Broadhurst , and J. R. C. von Holdt . 2026. “Spatial Framework for Assessing Chronic Containment Risks in Tailings Storage Facilities: A Case Study From South Africa.” Cleaner Waste Systems 11: 100536. 10.1016/j.clwas.2026.100536.

[crf370674-bib-0101] Koti, K. , A. Rodas‐Gonzalez , C. Nadon , T. McAllister , X. Yang , and C. Narváez‐Bravo . 2024. “Evaluating Disinfectant Efficacy on Mixed Biofilms Comprising Shiga Toxigenic *Escherichia coli*, Lactic Acid Bacteria, and Spoilage Microorganisms.” Frontiers in Microbiology 15: 1360645. 10.3389/fmicb.2024.1360645. PMC11021663 38633705

[crf370674-bib-0104] Krüger, G. I. , F. Urbina , C. Pardo‐Esté , et al. 2025. “Resilient by Design: Environmental Stress Promotes Biofilm Formation and Multi‐Resistance in Poultry‐Associated *Salmonella* .” Microorganisms 13, no. 8: 1812. 10.3390/microorganisms13081812. PMC12388525 40871316

[crf370674-bib-0105] Kumar, A. , S. Das , S. Ali , et al. 2025. “Mechanisms, Applications and Challenges of Natural Antimicrobials in Food System.” Food Bioscience 74: 107864. 10.1016/j.fbio.2025.107864.

[crf370674-bib-0106] Kumar, G. , S. Kumar , H. Jangid , J. Dutta , and A. Shidiki . 2025. “The Rise of Non‐Typhoidal *Salmonella*: An Emerging Global Public Health Concern.” Frontiers in Microbiology 16: 1524287. 10.3389/fmicb.2025.1524287. PMC11832534 39967739

[crf370674-bib-0107] Kunnath, A. P. , M. Suodha Suoodh , D. K. Chellappan , J. Chellian , and K. Palaniveloo . 2024. “Bacterial Persister Cells and Development of Antibiotic Resistance in Chronic Infections: An Update.” British Journal of Biomedical Science 81: 12958. 10.3389/bjbs.2024.12958. PMC11335562 39170669

[crf370674-bib-0108] Kuppusamy, S. , M. Meivelu , L. Praburaman , M. Mujahid Alam , A. G. Al‐Sehemi , and A. K . 2024. “Integrating AI in Food Contaminant Analysis: Enhancing Quality and Environmental Protection.” Journal of Hazardous Materials Advances 16: 100509. 10.1016/j.hazadv.2024.100509.

[crf370674-bib-0109] La, T.‐M. , T. Kim , H.‐J. Lee , et al. 2021. “Whole‐Genome Analysis of Multidrug‐Resistant *Salmonella* Enteritidis Strains Isolated From Poultry Sources in Korea.” Pathogens 10, no. 12: 1615. 10.3390/pathogens10121615. PMC8707440 34959570

[crf370674-bib-0110] Laconi, A. , R. Tolosi , C. Chirollo , et al. 2025. “From Farm to Slaughter: Tracing Antimicrobial Resistance in a Poultry Short Food Chain.” Antibiotics 14, no. 6: 604. 10.3390/antibiotics14060604. PMC12190163 40558194

[crf370674-bib-0111] Lamichhane, B. , A. M. M. Mawad , M. Saleh , et al. 2024. “Salmonellosis: An Overview of Epidemiology, Pathogenesis, and Innovative Approaches to Mitigate the Antimicrobial Resistant Infections.” Antibiotics 13, no. 1: 76. 10.3390/antibiotics13010076. PMC10812683 38247636

[crf370674-bib-0112] Li, H. , D. Fan , X. Liu , et al. 2026. “Stage‐Specific Therapeutic Strategies for Combating Bacterial Biofilm Infections: Recent Advances and Future Perspectives.” Journal of Controlled Release 393: 114815. 10.1016/j.jconrel.2026.114815. 41831689

[crf370674-bib-0113] Li, M. , G. Zhao , and M. M. Li . 2026. “Regulatory Mechanisms of Quorum Sensing in Microbial Communities and Their Potential Applications in Ruminant Livestock Production.” Journal of Advanced Research 83: 37–50. 10.1016/j.jare.2025.07.055. PMC13131436 40749791

[crf370674-bib-0114] Li, Y. , C. Barnett‐Neefs , and M. J. Stasiewicz . 2026. “Salmonellosis Risk Assessment for Comminuted Turkey Under Different Specificities of Concentration‐Based and Virulence‐Based Final Product Standards.” Microbial Risk Analysis 31: 100359. 10.1016/j.mran.2025.100359.

[crf370674-bib-0115] Liao, X. , Y. Ma , E. B.‐M. Daliri , et al. 2020. “Interplay of Antibiotic Resistance and Food‐Associated Stress Tolerance in Foodborne Pathogens.” Trends in Food Science & Technology 95: 97–106. 10.1016/j.tifs.2019.11.006.

[crf370674-bib-0116] Linn, K. Z. , S. Z. C. Lwin , A. T. Maung , et al. 2026. “Characterization, Virulent‐Determinants, Antimicrobial Resistance, and MALDI‐TOF MS Proteomic Profile of Nontyphoidal *Salmonella* Isolated From Chicken Meat in Fukuoka, Japan.” Microbiology Research 17, no. 3: 63. 10.3390/microbiolres17030063.

[crf370674-bib-0117] Liu, H. Y. , E. L. Prentice , and M. A. Webber . 2024. “Mechanisms of Antimicrobial Resistance in Biofilms.” npj Antimicrobials and Resistance 2, no. 1: 27. 10.1038/s44259-024-00046-3. PMC11445061 39364333

[crf370674-bib-0118] Liu, L. , G. Kong , Y. Yuan , Z. Ji , X. Wu , and C. Chang . 2026. “Pilot‐Scale Study and Treatment Performance Prediction of Oilfield‐Produced Water Using Superoleophobic Fiber Coalescence Technology.” Process Safety and Environmental Protection 207: 108365. 10.1016/j.psep.2025.108365.

[crf370674-bib-0119] Lu, Y. , B. Zhang , Z. Wang , et al. 2026. “From Host‐Derived Pressures to the Environmental Anti‐Antimicrobial Peptides Resistome: Mechanisms, Reservoirs and Implications for Therapeutic Peptide Design.” Marine Drugs 24, no. 2: 76. 10.3390/md24020076. PMC12942635 41745479

[crf370674-bib-0120] Magdalena, A. , S. Agún , J. Álvarez , L. Fernández , and P. García . 2026. “Biofilms in Food Processing: Rethinking Disinfection Strategies in the Era of Resistance.” Current Opinion in Food Science 70: 101416. 10.1016/j.cofs.2026.101416.

[crf370674-bib-0121] Majumdar, A. , D. Bagchi , I. Kotta‐Loizou , and M. Buck . 2026. “The One Health Resistome: Integrating Environmental, Microbial, and Human Antimicrobial Resistance Surveillance and Risk Analysis in the Digital Age.” Journal of Hazardous Materials 513: 142431. 10.1016/j.jhazmat.2026.142431. 42208292

[crf370674-bib-0122] Makovska, I. , E. Biebaut , P. Dhaka , et al. 2025. “Methods for Assessing Efficacy of Cleaning and Disinfection in Livestock Farms: A Narrative Review.” Frontiers in Veterinary Science 12: 1581217. 10.3389/fvets.2025.1581217. PMC12400523 40901067

[crf370674-bib-0123] Marcu, D. , I. Balta , M. Harvey , et al. 2026a. “Impact of Farm Management Practices on *Salmonella* Occurrence at the Farm Level—A Blend of Traditional Methods and Artificial Intelligence.” Foods 15, no. 4: 676. 10.3390/foods15040676. PMC12939011 41750866

[crf370674-bib-0124] Marcu, D. , I. Balta , M. Harvey , et al. 2026b. “Strategies to Manage *Salmonella* in Meat, Egg, and Dairy Processing Plants.” Journal of Food Protection 89, no. 3: 100708. 10.1016/j.jfp.2026.100708. 41617087

[crf370674-bib-0125] Marin, C. , M. Cerdà‐Cuéllar , S. González‐Bodi , L. Lorenzo‐Rebenaque , and S. Vega . 2022. “Research Note: Persistent *Salmonella* Problems in Slaughterhouses Related to Clones Linked to Poultry Companies.” Poultry Science 101, no. 8: 101968. 10.1016/j.psj.2022.101968. PMC9249845 35759998

[crf370674-bib-0126] Marmion, M. , G. Macori , M. Ferone , P. Whyte , and A. G. M. Scannell . 2022. “Survive and Thrive: Control Mechanisms That Facilitate Bacterial Adaptation to Survive Manufacturing‐Related Stress.” International Journal of Food Microbiology 368: 109612. 10.1016/j.ijfoodmicro.2022.109612. 35278797

[crf370674-bib-0127] Maroju, P. A. , A. S. Sidhu , A. R. Motaganahalli , et al. 2026. “Detection to Disruption: A Comprehensive Review of Bacterial Biofilms and Therapeutic Advances.” Antibiotics 15, no. 4: 396. 10.3390/antibiotics15040396. PMC13113663 42041359

[crf370674-bib-0128] Marshall, K. E. , Z. Cui , B. L. Gleason , et al. 2024. “An Approach to Describe *Salmonella* Serotypes of Concern for Outbreaks: Using Burden and Trajectory of Outbreak‐Related Illnesses Associated With Meat and Poultry.” Journal of Food Protection 87, no. 4: 100331. 10.1016/j.jfp.2024.100331. PMC11366486 39032785

[crf370674-bib-0129] Mekonnen, G. B. 2026. “Integrative Multi‐Omics and Predictive Precision Systems for Poultry Meat and Egg Quality: Mechanisms, Applications, and Commercial Challenges.” Poultry Science 105, no. 10: 107378. 10.1016/j.psj.2026.107378. PMC13380483 42435607

[crf370674-bib-0052] de Melo, R. T. , T. dos Reis Cardoso , P. A. Peres , R. F. Braz , G. P. Monteiro , and D. A. Rossi . 2021. “ *Salmonella enterica* Serovar Minnesota Biofilms, Susceptibility to Biocides and Molecular Characterization.” Pathogens 10, no. 5: 581. 10.3390/pathogens10050581. PMC8150743 34064554

[crf370674-bib-0130] Mendoza, I. C. , E. O. Luna , M. D. Pozo , et al. 2022. “Conventional and Non‐Conventional Disinfection Methods to Prevent Microbial Contamination in Minimally Processed Fruits and Vegetables.” LWT ‐ Food Science and Technology 165: 113714. 10.1016/j.lwt.2022.113714. PMC9239846 35783661

[crf370674-bib-0131] Merino, L. , F. Procura , F. M. Trejo , D. J. Bueno , and M. A. Golowczyc . 2019. “Biofilm Formation by *Salmonella* sp. in the Poultry Industry: Detection, Control and Eradication Strategies.” Food Research International 119: 530–540. 10.1016/j.foodres.2017.11.024. 30884686

[crf370674-bib-0132] Metris, A. , A. W. Walker , A. Showering , et al. 2025. “Assessing the Safety of Microbiome Perturbations.” Microbial Genomics 11, no. 5: 001405. 10.1099/mgen.0.001405. PMC12282321 40371892

[crf370674-bib-0133] Michaelis, C. , and E. Grohmann . 2023. “Horizontal Gene Transfer of Antibiotic Resistance Genes in Biofilms.” Antibiotics 12, no. 2: 328. 10.3390/antibiotics12020328. PMC9952180 36830238

[crf370674-bib-0134] Mirghani, R. , T. Saba , H. Khaliq , et al. 2022. “Biofilms: Formation, Drug Resistance and Alternatives to Conventional Approaches.” AIMS Microbiology 8, no. 3: 239–277. 10.3934/microbiol.2022019. PMC9576500 36317001

[crf370674-bib-0135] Mishra, S. , A. Gupta , V. Upadhye , S. C. Singh , R. P. Sinha , and D.‐P. Häder . 2023. “Therapeutic Strategies Against Biofilm Infections.” Life 13, no. 1: 172. 10.3390/life13010172. PMC9866932 36676121

[crf370674-bib-0136] Mitra, A. , and S. Mukhopadhyay . 2023. “Regulation of Biofilm Formation by Non‐Coding RNA in Prokaryotes.” Current Research in Pharmacology and Drug Discovery 4: 100151. 10.1016/j.crphar.2022.100151. PMC9829692 36636617

[crf370674-bib-0137] Mitra, S. , R. Verma , A. Singh , R. K. Saran , and S. Lazar . 2026. “Pesticide Remediation in Soil and Aquatic Systems: Comparative Assessment of Conventional and Advanced Treatment Pathways.” Next Sustainability 7: 100281. 10.1016/j.nxsust.2026.100281.

[crf370674-bib-0138] Mkangara, M. 2023. “Prevention and Control of Human *Salmonella enterica* Infections: An Implication in Food Safety.” International Journal of Food Science 2023: 8899596. 10.1155/2023/8899596. PMC10506869 37727836

[crf370674-bib-0139] Muneeb, M. , E. Ullah Khan , S. Ahmad , et al. 2025. “A Comprehensive Review of the Application of Bacteriophages Against Enteric Bacterial Infection in Poultry: Current Status, Challenges, and Future Prospects.” Antibiotics 14, no. 12: 1207. 10.3390/antibiotics14121207. PMC12729848 41463709

[crf370674-bib-0140] Murugaiyan, J. , P. A. Kumar , G. S. Rao , et al. 2022. “Progress in Alternative Strategies to Combat Antimicrobial Resistance: Focus on Antibiotics.” Antibiotics 11, no. 2: 200. 10.3390/antibiotics11020200. PMC8868457 35203804

[crf370674-bib-0141] Murugesan, T. , S. Pugazhendhi , M. Muthukumar , K. Arumugam , and R. Sehgal . 2026. “Decoding Biofilm Tolerance in Clinical Infections: Matrixome Architecture, Metabolic Zonation, and Therapeutic Implications.” The Microbe 11: 100753. 10.1016/j.microb.2026.100753.

[crf370674-bib-0142] Musa, L. , V. Toppi , V. Stefanetti , et al. 2024. “High Biofilm‐Forming Multidrug‐Resistant *Salmonella* Infantis Strains From the Poultry Production Chain.” Antibiotics 13, no. 7: 595. 10.3390/antibiotics13070595. PMC11273867 39061277

[crf370674-bib-0143] Muteeb, G. , R. N. A. Kazi , M. Aatif , A. Azhar , M. E. Oirdi , and M. Farhan . 2025. “Antimicrobial Resistance: Linking Molecular Mechanisms to Public Health Impact.” SLAS Discovery 33: 100232. 10.1016/j.slasd.2025.100232. 40216324

[crf370674-bib-0145] Neelawala, R. N. , L. K. Edison , and S. Kariyawasam . 2024. “Pre‐Harvest Non‐Typhoidal *Salmonella* Control Strategies in Commercial Layer Chickens.” Animals 14, no. 24: 3578. 10.3390/ani14243578. PMC11672659 39765482

[crf370674-bib-0146] Nour El‐Din, H. T. , A. S. Yassin , Y. M. Ragab , and A. M. Hashem . 2021. “Phenotype‐Genotype Characterization and Antibiotic‐Resistance Correlations Among Colonizing and Infectious Methicillin‐Resistant *Staphylococcus aureus* Recovered From Intensive Care Units.” Infection and Drug Resistance 14: 1557–1571. 10.2147/idr.s296000. PMC8071083 33907431

[crf370674-bib-0147] Obe, T. , A. S. Kiess , and R. Nannapaneni . 2024. “Antimicrobial Tolerance in *Salmonella*: Contributions to Survival and Persistence in Processing Environments.” Animals 14, no. 4: 578. 10.3390/ani14040578. PMC10886206 38396546

[crf370674-bib-0148] Obe, T. , R. Nannapaneni , W. Schilling , L. Zhang , and A. Kiess . 2021. “Antimicrobial Tolerance, Biofilm Formation, and Molecular Characterization of *Salmonella* Isolates From Poultry Processing Equipment.” Journal of Applied Poultry Research 30, no. 4: 100195. 10.1016/j.japr.2021.100195.

[crf370674-bib-0149] Oliveira, M. M. , D. P. Amorim‐Neto , and A. S. Sant'Ana . 2026. “How *Salmonella* Works Under Osmotic and Desiccation Stresses.” Comprehensive Reviews in Food Science and Food Safety 25, no. 3: e70439. 10.1111/1541-4337.70439. PMC13033924 41906447

[crf370674-bib-0150] Pal, M. K. , and M. Lavanya . 2022. “Microbial Influenced Corrosion: Understanding Bioadhesion and Biofilm Formation.” Journal of Bio‐ and Tribo‐Corrosion 8, no. 3: 76. 10.1007/s40735-022-00677-x.

[crf370674-bib-0151] Pan, M. , H. Li , X. Han , et al. 2022. “Effects of Hydrodynamic Conditions on the Composition, Spatiotemporal Distribution of Different Extracellular Polymeric Substances and the Architecture of Biofilms.” Chemosphere 307: 135965. 10.1016/j.chemosphere.2022.135965. 35963380

[crf370674-bib-0152] Pang, X. , X. Hu , X. Du , C. Lv , and H.‐G. Yuk . 2023. “Biofilm Formation in Food Processing Plants and Novel Control Strategies to Combat Resistant Biofilms: The Case of *Salmonella* spp.” Food Science and Biotechnology 32, no. 12: 1703–1718. 10.1007/s10068-023-01349-3. PMC10533767 37780596

[crf370674-bib-0153] Peters, M. D. J. , C. Marnie , A. C. Tricco , et al. 2020. “Updated Methodological Guidance for the Conduct of Scoping Reviews.” JBI Evidence Synthesis 18, no. 10: 2119–2126. 10.11124/jbies-20-00167. 33038124

[crf370674-bib-0154] Pettengill, J. B. , H. Rand , S. S. Wang , D. Kautter , A. Pightling , and Y. Wang . 2022. “Transient and Resident Pathogens: Intra‐Facility Genetic Diversity of *Listeria monocytogenes* and *Salmonella* From Food Production Environments.” PLoS ONE 17, no. 9: e0268470. 10.1371/journal.pone.0268470. PMC9436056 36048885

[crf370674-bib-0155] Pradhan, D. , and V. Devi Negi . 2019. “Stress‐Induced Adaptations in *Salmonella*: A Ground for Shaping Its Pathogenesis.” Microbiological Research 229: 126311. 10.1016/j.micres.2019.126311. 31446332

[crf370674-bib-0156] Punchihewage‐Don, A. J. , J. Hawkins , A. M. Adnan , F. Hashem , and S. Parveen . 2022. “The Outbreaks and Prevalence of Antimicrobial Resistant *Salmonella* in Poultry in the United States: An Overview.” Heliyon 8, no. 11: e11571. 10.1016/j.heliyon.2022.e11571. PMC9668525 36406693

[crf370674-bib-0157] Radu, E. , A. Dima , E. M. Dobrota , et al. 2023. “Global Trends and Research Hotspots on HACCP and Modern Quality Management Systems in the Food Industry.” Heliyon 9, no. 7: e18232. 10.1016/j.heliyon.2023.e18232. PMC10393635 37539220

[crf370674-bib-0158] Rapacka‐Zdonczyk, A. 2026. “Beyond Resistance: Phenotypic Plasticity in Bacterial Responses to Antibiotics, Oxidative Stress and Antimicrobial Photodynamic Inactivation.” Molecules 31, no. 3: 567. 10.3390/molecules31030567. PMC12899298 41683545

[crf370674-bib-0159] Rather, M. A. , K. Gupta , and M. Mandal . 2021. “Microbial Biofilm: Formation, Architecture, Antibiotic Resistance, and Control Strategies.” Brazilian Journal of Microbiology 52, no. 4: 1701–1718. 10.1007/s42770-021-00624-x. PMC8578483 34558029

[crf370674-bib-0160] Reem, C. S. A. , M. A. H. Chowdhury , M. D. Ashrafudoulla , and S. D. Ha . 2025. “Leveraging Blockchain and AI for Biofilm Control in Food Processing Environments.” Comprehensive Reviews in Food Science and Food Safety 24, no. 5: e70261. 10.1111/1541-4337.70261. 40843956

[crf370674-bib-0161] Rhouma, M. , P. Romero‐Barrios , M.‐L. Gaucher , and S. Bhachoo . 2020. “Antimicrobial Resistance Associated With the Use of Antimicrobial Processing Aids During Poultry Processing Operations: Cause for Concern?” Critical Reviews in Food Science and Nutrition 61, no. 19: 3279–3296. 10.1080/10408398.2020.1798345. 32744054

[crf370674-bib-0162] Rocha, J. S. , B. M. Lepaus , M. M. Domingos , P. C. Bernardes , and J. F. de São José . 2026. “Study of the Factors Involved in the Adhesion Process of *Salmonella enterica* Enteritidis, *Escherichia coli*, and *Staphylococcus aureus* to the Surface of Apple, Arugula, Cucumber, and Strawberry.” Foods 15, no. 3: 449. 10.3390/foods15030449. PMC12896527 41683037

[crf370674-bib-0163] Romero, M. , J. Luckett , J.‐F. Dubern , et al. 2025. “Combinatorial Discovery of Microtopographical Landscapes That Resist Biofilm Formation Through Quorum Sensing Mediated Autolubrication.” Nature Communications 16, no. 1: 5295. 10.1038/s41467-025-60567-x. PMC12177056 40533450

[crf370674-bib-0164] Rossi, D. A. , R. T. Melo , E. P. Mendonça , and G. P. Monteiro . 2017. “Biofilms of *Salmonella* and Campylobacter in the Poultry Industry.” In Poultry Science, edited by M. Manafi . IntechOpen. 10.5772/65254.

[crf370674-bib-0165] Roy, S. , G. Chowdhury , A. K. Mukhopadhyay , S. Dutta , and S. Basu . 2022. “Convergence of Biofilm Formation and Antibiotic Resistance in *Acinetobacter baumannii* Infection.” Frontiers in Medicine 9: 793615. 10.3389/fmed.2022.793615. PMC8987773 35402433

[crf370674-bib-0166] Rubrice, R. , V. Gueneau , R. Briandet , A. Cornuejols , and V. Guigue . 2025. “A Machine Learning Framework for the Prediction and Analysis of Bacterial Antagonism in Biofilms Using Morphological Descriptors.” Artificial Intelligence in the Life Sciences 8: 100137. 10.1016/j.ailsci.2025.100137.

[crf370674-bib-0167] Rudhart, S. A. , F. Günther , L. Dapper , et al. 2020. “UV Light‐Based Decontamination: An Effective and Fast Way for Disinfection of Endoscopes in Otorhinolaryngology?” European Archives of Oto‐Rhino‐Laryngology 277, no. 8: 2363–2369. 10.1007/s00405-020-05978-w. PMC7335372 32358650

[crf370674-bib-0168] Rugarabamu, S. , and G. Mwanyika . 2025. “Biotechnological Innovations to Combat Antimicrobial Resistance and Advance Global Health Equity.” Bacteria 4, no. 3: 46. 10.3390/bacteria4030046.

[crf370674-bib-0169] Sakalauskienė, G. V. , and A. Radzevičienė . 2025. “Biofilm and Outer Membrane Vesicle Formation in ESKAPE Gram‐Negative Bacteria: A Comprehensive Review.” International Journal of Molecular Sciences 26, no. 20: 9857. 10.3390/ijms26209857. PMC12563647 41155151

[crf370674-bib-0170] Sanches, P. H. G. , N. C. de Melo , A. M. Porcari , and L. M. de Carvalho . 2024. “Integrating Molecular Perspectives: Strategies for Comprehensive Multi‐Omics Integrative Data Analysis and Machine Learning Applications in Transcriptomics, Proteomics and Metabolomics.” Biology 13, no. 1: 848. 10.3390/biology13110848. PMC11592251 39596803

[crf370674-bib-0171] Shaheed, I. , A. Samir , E. Ismael , et al. 2026. “Virulence Determinants and Multidrug Resistance of Emerging *Salmonella enterica* Subspecies Salamae in Broiler Chickens: A Potential Zoonotic Concern.” Veterinary Research Communications 50, no. 4: 273. 10.1007/s11259-026-11163-x. PMC13090236 41995952

[crf370674-bib-0172] Sharif, S. , and A. K. Yadav . 2025. “Bacterial Biofilm and Its Role in Antibiotic Resistance.” The Microbe 7: 100356. 10.1016/j.microb.2025.100356.

[crf370674-bib-0173] Shi, Y. , H. Sanderson , J. Chuan , et al. 2026. “Dual‐Platform Metagenomic Surveillance Distinguishes Pathogen and Resistome Hotspots Across Agricultural and Mixed‐Use Watersheds.” One Health 22: 101384. 10.1016/j.onehlt.2026.101384. PMC13015239 41890980

[crf370674-bib-0175] Siceloff, A. T. , D. Waltman , C. E. Gunning , S. P. Nolan , P. Rohani , and N. W. Shariat . 2025. “Longitudinal Study Highlights Patterns of *Salmonella* Serovar Co‐Occurrence and Exclusion in Commercial Poultry Production.” Frontiers in Microbiology 16: 1570593. 10.3389/fmicb.2025.1570593. PMC12307445 40740319

[crf370674-bib-0176] Sidky, M. B. , U. H. Issa , M. S. Hassan , and M. R. O. Ali . 2026. “Analysis of Risks Affecting the Operation and Maintenance of Wastewater Treatment Plant Systems.” Journal of Engineering and Applied Science 73, no. 1: 27. 10.1186/s44147-025-00786-7.

[crf370674-bib-0177] Singh, A. K. , R. Gautam , and G. Manik . 2026. “Versatile Applications of EPS Secreting *Microbes*: Advances in Microbial Fuel Cells and Beyond.” Biotechnology for Sustainable Materials 3: 4. 10.1186/s44316-026-00053-6.

[crf370674-bib-0178] Singh, V. , S. Sinha , and J. Verma . 2026. “Bioinformatics Models in Drug Delivery: Predicting Biomaterial‐Biological Interactions for Targeted Therapies.” Next Nanotechnology 9: 100335. 10.1016/j.nxnano.2025.100335.

[crf370674-bib-0179] Sohail, M. N. , S. Isloor , D. Rathnamma , et al. 2026. “Profiling of Antimicrobial Resistance Genes and Genotype–Phenotype Associations in *Salmonella* spp. Isolated Along the Broiler Production Chain in Karnataka, India.” International Journal of Food Science 2026, no. 1: 8152167. 10.1155/ijfo/8152167. PMC13126426 42064423

[crf370674-bib-0180] Sophian, A. , W. Yulianto , and F. U. Syahadat . 2026. “Investigating Foodborne Pathogen Outbreaks: An Integrated Framework for Tracing, Detection, and Risk Assessment in Food Systems.” Exploration of Foods and Foodomics 4: 1010140. 10.37349/eff.2026.1010140.

[crf370674-bib-0181] Su, Q. , Y. Xue , C. Wang , et al. 2025. “Strategies and Applications of Antibacterial Surface‐Modified Biomaterials.” Bioactive Materials 53: 114–140. 10.1016/j.bioactmat.2025.07.009. PMC12274879 40688018

[crf370674-bib-0182] Subramanian, I. , S. Verma , S. Kumar , A. Jere , and K. Anamika . 2020. “Multi‐Omics Data Integration, Interpretation, and Its Application.” Bioinformatics and Biology Insights 14: 1–24. 10.1177/1177932219899051. PMC7003173 32076369

[crf370674-bib-0183] Tavaddod, S. , A. Dawson , and R. J. Allen . 2024. “Bacterial Aggregation Triggered by Low‐Level Antibiotic‐Mediated Lysis.” npj Biofilms and Microbiomes 10, no. 1: 90. 10.1038/s41522-024-00553-1. PMC11427687 39327479

[crf370674-bib-0184] Ugwu, C. N. , E. N. Ezeibe , S. C. Emencheta , et al. 2025. “Biofilms: Structure, Resistance Mechanism, Emerging Control Strategies, and Applications.” RSC Pharmaceutics 2: 1376–1407. 10.1039/D5PM00094G.

[crf370674-bib-0185] Uruén, C. , G. Chopo‐Escuin , J. Tommassen , R. C. Mainar‐Jaime , and J. Arenas . 2021. “Biofilms as Promoters of Bacterial Antibiotic Resistance and Tolerance.” Antibiotics 10, no. 1: 3. 10.3390/antibiotics10010003. PMC7822488 33374551

[crf370674-bib-0065] USDA Food Safety and Inspection Service (FSIS) . 2021. “Performance Standards: *Salmonella* Verification Program for Raw Poultry Products (FSIS Directive 10250.2).” US Department of Agriculture. https://www.fsis.usda.gov/policy/fsis‐directives/10250.2.

[crf370674-bib-0186] USDA Food Safety and Inspection Service (FSIS) . 2013. “Salmonella by the Numbers.” https://www.fsis.usda.gov/inspection/inspection‐programs/inspection‐poultry‐products/reducing‐salmonella‐poultry/salmonella.

[crf370674-bib-0187] Valde, D. , T. Kumar , and S. E. Jujjavarapu . 2026. “Real‐Time Sensing Approaches and Antibiofilm Strategies for Biofilm‐Associated Infections: Advances and Perspectives on Medical Devices and Clinical Contexts.” Future Journal of Pharmaceutical Sciences 12, no. 1: 25. 10.1186/s43094-026-00944-5.

[crf370674-bib-0188] Vice, Z. , Y. Zhou , S. Chitlapilly Dass , and R. Wang . 2025. “Microscopic Analysis of Temperature Effects on Surface Colonization and Biofilm Morphology of *Salmonella enterica* .” Foods 14, no. 2: 268. 10.3390/foods14020268. PMC11764560 39856935

[crf370674-bib-0189] Vo, H. H. , T. T. Le , T. V. Nguyen , et al. 2026. “Developing an Optimized Method for Biofilm Extraction From Microplastic Surfaces for High‐Efficiency Analysis of Adherent Bacterial Communities.” Applied and Environmental Microbiology 92, no. 5: e00416. 10.1128/aem.00416-26. PMC13188912 42053316

[crf370674-bib-0190] Vohra, M. , A. Kour , N. P. Kalia , et al. 2024. “A Comprehensive Review of Genomics, Transcriptomics, Proteomics, and Metabolomic Insights Into the Differentiation of *Pseudomonas aeruginosa* From the Planktonic to Biofilm State: A Multi‐Omics Approach.” International Journal of Biological Macromolecules 257: 128563. 10.1016/j.ijbiomac.2023.128563. 38070800

[crf370674-bib-0191] Wang, C. , X. Wang , J. Hao , et al. 2024. “Serotype Distribution and Antimicrobial Resistance of *Salmonella* Isolates From Poultry Sources in China.” Antibiotics 13, no. 10: 959. 10.3390/antibiotics13100959. PMC11503990 39452225

[crf370674-bib-0192] Wang, D. , G. C. Fletcher , S. L. W. On , J. S. Palmer , D. Gagic , and S. H. Flint . 2023. “Biofilm Formation, Sodium Hypochlorite Susceptibility and Genetic Diversity of Vibrio Parahaemolyticus.” International Journal of Food Microbiology 385: 110011. 10.1016/j.ijfoodmicro.2022.110011. 36370527

[crf370674-bib-0193] Wang, D. , L. Wang , Q. Liu , and Y. Zhao . 2025. “Virulence Factors in Biofilm Formation and Therapeutic Strategies for *Staphylococcus aureu*s: A Review.” Animals and Zoonoses 1, no. 2: 188–202. 10.1016/j.azn.2024.11.003.

[crf370674-bib-0194] Wang, J. , I. Hashem , S. Bhonsale , and J. F M Van Impe . 2025. “Individual‐Based Modeling Unravels Spatial and Social Interactions in Bacterial Communities.” The ISME Journal 19, no. 1: wraf116. 10.1093/ismejo/wraf116. PMC12411854 40838740

[crf370674-bib-0195] Wang, X. , M. Liu , C. Yu , J. Li , and X. Zhou . 2023. “Biofilm Formation: Mechanistic Insights and Therapeutic Targets.” Molecular Biomedicine 4: 49. 10.1186/s43556-023-00164-w. PMC10721784 38097907

[crf370674-bib-0196] Wang, X. , G. Zhao , X. Qu , X. Yang , J. Wang , and P. Wang . 2023. “Influence of Cooling Water Parameters on the Thermal Performance of the Secondary Circuit System of a Modular High‐Temperature Gas‐Cooled Reactor Nuclear Power Plant.” Energies 16, no. 18: 6560. 10.3390/en16186560.

[crf370674-bib-0197] Wang, Z. , M. Wang , B. Dong , Y. Wang , Z. Ding , and S. Shen . 2025. “Drug‐Tolerant Persister Cells in Cancer: Bridging the Gaps Between Bench and Bedside.” Nature Communications 16, no. 1: 10048. 10.1038/s41467-025-66376-6. PMC12623778 41249194

[crf370674-bib-0198] Wenger, C. , S. Oeljeklaus , B. Warscheid , A. Schneider , and A. Harsman . 2017. “A Trypanosomal Orthologue of an Intermembrane Space Chaperone Has a Non‐Canonical Function in Biogenesis of the Single Mitochondrial Inner Membrane Protein Translocase.” PLoS Pathogens 13, no. 8: e1006550. 10.1371/journal.ppat.1006550. PMC5584982 28827831

[crf370674-bib-0199] Willson, N. L. , and K. Chousalkar . 2023. “Dominant *Salmonella* Serovars in Australian Broiler Breeder Flocks and Hatcheries: A Longitudinal Study.” Applied and Environmental Microbiology 89, no. 8: e00627. 10.1128/aem.00627-23. PMC10467335 37466445

[crf370674-bib-0200] Xu, J. , J. Wang , Z. Kang , et al. 2025. “Mixed‐Species Biofilm With *Salmonella* in Food Industry: Persistence, Interspecies Interaction, and Control.” Food Research International 221: 117348. 10.1016/j.foodres.2025.117348. 41174425

[crf370674-bib-0201] Xue, H. , L. Liu , L. Liu , et al. 2026. “Intelligent Design and Application of Molecular Recognition Hydrogels in Tissue Engineering.” Materials Today Bio 38: 103048. 10.1016/j.mtbio.2026.103048. PMC13049675 41938135

[crf370674-bib-0206] Yücetepe, A. , Z. S. Güneş , N. Adrar , and T. Esatbeyoglu . 2026. “Non‐Thermal Processing of Plant‐Based Proteins: Comparison of Cold Plasma Treatment With High Hydrostatic Pressure, Ultraviolet Radiation and Ultrasonication Treatments.” European Food Research and Technology 252: 143. 10.1007/s00217-026-05071-2.

[crf370674-bib-0202] Yaki, L. M. 2026. “Bacterial Biofilms and Antimicrobial Resistance: A Global Threat.” Discover Bacteria 3, no. 1: 15. 10.1007/s44351-025-00035-5.

[crf370674-bib-0203] Yang, S. , J. Fan , L. Yu , et al. 2024. “Dissemination of Ceftriaxone‐Resistant *Salmonella* Enteritidis Harboring Plasmids Encoding blaCTX‐M‐55 or blaCTX‐M‐14 Gene in China.” Antibiotics 13, no. 5: 456. 10.3390/antibiotics13050456. PMC11117602 38786182

[crf370674-bib-0204] Yogendraiah, K. M. , B. Sadanandan , L. K. Natraj , V. Vijayalakshmi , and K. Shetty . 2026. “Biofilm Formation Directly Correlates With Cell Viability in *Candida tropicalis* on Polypropylene.” Applied Microbiology and Biotechnology 110, no. 1: 13758. 10.1007/s00253-026-13758-5. PMC12960443 41779039

[crf370674-bib-0205] Yu, D. , J.‐E. Lee , M.‐S. Rhee , et al. 2025. “Isolation of Biofilm‐Forming Bacteria From Food Processing Equipment Surfaces and the Biofilm‐Degrading Activity of *Bacillus licheniformis* YJE5.” Foods 14, no. 21: 3592. 10.3390/foods14213592. PMC12607818 41227566

[crf370674-bib-0207] Zeng, S. , L. Tariq , Y. Bi , M. Guo , H. Wang , and F. Song . 2025. “PLATZ Transcription Factors: Roles in Plant Growth, Development, and Stress Adaptation.” Plant Stress 18: 101075. 10.1016/j.stress.2025.101075.

[crf370674-bib-0208] Zhang, X. , Z. Dong , S. Zhang , J. Ma , and S. Liu . 2025. “Microplastic Biofilm as Hotspots of Antibiotic Resistance Genes and Potential Pathogens.” npj Biofilms and Microbiomes 12, no. 1: 24. 10.1038/s41522-025-00890-9. PMC12847985 41429800

[crf370674-bib-0209] Zhao, A. , J. Sun , and Y. Liu . 2023. “Understanding Bacterial Biofilms: From Definition to Treatment Strategies.” Frontiers in Cellular and Infection Microbiology 13: 1137947. 10.3389/fcimb.2023.1137947. PMC10117668 37091673

[crf370674-bib-0210] Zhou, T. , J. Chen , Z. Kang , et al. 2026. “Microplastic Biofilms Enhance Environmental Stress, Disinfectant, and Antibiotic Tolerance of *Salmonella enterica* Serovar Typhimurium in Simulated Fresh Vegetable Processing Systems.” Food Research International 236: 119183. 10.1016/j.foodres.2026.119183. 42116460

